# Palbociclib-based high-throughput combination drug screening identifies synergistic therapeutic options in HPV-negative head and neck squamous cell carcinoma

**DOI:** 10.1186/s12916-022-02373-6

**Published:** 2022-05-12

**Authors:** Ziyue Gu, Chaoji Shi, Jiayi Li, Yong Han, Bao Sun, Wuchang Zhang, Jing Wu, Guoyu Zhou, Weimin Ye, Jiang Li, Zhiyuan Zhang, Rong Zhou

**Affiliations:** 1 Department of Oral and Maxillofacial-Head Neck Oncology, Shanghai Ninth People’s Hospital, College of Stomatology, Shanghai Jiao Tong University School of Medicine, Shanghai, 200011 China; 2 National Clinical Research Center for Oral Diseases, Shanghai, 200011 China; 3 Shanghai Key Laboratory of Stomatology & Shanghai Research Institute of Stomatology, Shanghai, 200011 China; 4 Research Unit of Oral and Maxillofacial Regenerative Medicine, Chinese Academy of Medical Sciences, Shanghai, 200011 China; 5 Department of Oral Pathology, Ninth People’s Hospital, Shanghai Jiao Tong University, School of Medicine, Shanghai, 200011 China; 6 Laboratory of Oral Microbiota and Systemic Diseases Shanghai Ninth People’s Hospital, College of Stomatology, Shanghai Jiao Tong University School of Medicine, Shanghai, 200011 China

**Keywords:** HPV^neg^ HNSCC, Palbociclib, Combination drug screening, *PIK3CA*, Alpelisib, Patient-derived xenografts

## Abstract

**Background:**

Deregulation of cell-cycle pathway is ubiquitously observed in human papillomavirus negative (HPV^neg^) head and neck squamous cell carcinoma (HNSCC). Despite being an attractive target, CDK4/6 inhibition using palbociclib showed modest or conflicting results as monotherapy or in combination with platinum-based chemotherapy or cetuximab in HPV^neg^ HNSCC. Thus, innovative agents to augment the efficacy of palbociclib in HPV^neg^ HNSCC would be welcomed.

**Methods:**

A collection of 162 FDA-approved and investigational agents was screened in combinatorial matrix format, and top combinations were validated in a broader panel of HPV^neg^ HNSCC cell lines. Transcriptional profiling was conducted to explore the molecular mechanisms of drug synergy. Finally, the most potent palbociclib-based drug combination was evaluated and compared with palbociclib plus cetuximab or cisplatin in a panel of genetically diverse HPV^neg^ HNSCC cell lines and patient-derived xenograft models.

**Results:**

Palbociclib displayed limited efficacy in HPV^neg^ HNSCC as monotherapy. The high-throughput combination drug screening provided a comprehensive palbociclib-based drug-drug interaction dataset, whereas significant synergistic effects were observed when palbociclib was combined with multiple agents, including inhibitors of the PI3K, EGFR, and MEK pathways. PI3K pathway inhibitors significantly reduced cell proliferation and induced cell-cycle arrest in HPV^neg^ HNSCC cell lines when combined with palbociclib, and alpelisib (a PI3Kα inhibitor) was demonstrated to show the most potent synergy with particularly higher efficacy in HNSCCs bearing *PIK3CA* alterations. Notably, when compared with cisplatin and cetuximab, alpelisib exerted stronger synergism in a broader panel of cell lines. Mechanistically, RRM2-dependent epithelial mesenchymal transition (EMT) induced by palbociclib, was attenuated by alpelisib and cetuximab rather than cisplatin. Subsequently, PDX models with distinct genetic background further validated that palbociclib plus alpelisib had significant synergistic effects in models harboring *PIK3CA* amplification.

**Conclusions:**

This study provides insights into the systematic combinatory effect associated with CDK4/6 inhibition and supports further initiation of clinical trials using the palbociclib plus alpelisib combination in HPV^neg^ HNSCC with *PIK3CA* alterations.

**Supplementary Information:**

The online version contains supplementary material available at 10.1186/s12916-022-02373-6.

## Background

Human papillomavirus negative (HPV^neg^) head and neck squamous cell carcinoma (HNSCC) is a major subtype of HNSCC, which is associated with poorer clinical outcomes and lower response rate to chemo-radiotherapy as well as treatment targeting EGFR and PD-1 when compared to HPV-positive disease [1–5]. Deregulation of cell-cycle signaling mediated by *CCND1* and *CDKN2A* aberrations was observed in more than half of the HPV^neg^ HNSCCs [6]; a finding that strongly recommends the investigation of CDK4/6 inhibitors in this disease. Considering the mechanisms of CDK4/6 inhibition, profound effect that causing tumor regression was rarely observed when CDK4/6 inhibitor was used as a monotherapy in HNSCC [7, 8]. On the other hand, when combined with endocrine therapy, CDK4/6 inhibitor has become a first-line therapy as a combination drug with endocrine therapy for estrogen receptor (ER)-positive and HER2-negative breast cancer [9], and multiple clinical trials are exploring various combination treatments involving CDK4/6 inhibitors [10].

Palbociclib is the first FDA-approved CDK4/6 inhibitor which is currently under active clinical investigation as the combinational agent in HNSCC [11]. Currently, several phase I-II clinical trials are in progress to evaluate palbociclib in combination with cetuximab or platinum-based chemotherapies in HNSCC (NCT02499120, NCT02101034, NCT03024489, NCT03065062, and NCT03088059). Very recently, results from these trials showed conflicting findings regarding the therapeutic efficacy using combination of palbociclib plus cetuximab [12, 13], while combination of platinum-based chemotherapy plus palbociclib showed insufficient antitumor activity in recurrent/metastatic HNSCC patients [14]. Thus, rational combinations with palbociclib would fulfill the promise of targeting the cell-cycle pathway in HPV^neg^ HNSCC and could be more readily integrated to the current treatment regimens during clinical translation.

To circumvent potential drug resistance which is commonly observed using monotherapies and induce a synergistic treatment effect, drug combination strategy is currently the mainstream approach in cancer treatment [15]. More recently, systematic matrix screening of drug combinations using large-scale compound libraries offers a more preferred pipeline to rapidly identify robust single agent as well as assess the potential of candidate agents as synergistic combinations [16–18]. Additionally, unbiased matrix screening represents a high-throughput means to provide a roadmap of drug-drug pairs that are synergistic, additive, or antagonistic [19]. By using high-throughput drug screening method, multiple promising drug combinations are currently under clinical investigation, like NCT02756247, in which BKM120 (a PI3K signaling pathway inhibitor) was previously discovered that would cooperate with ibrutinib (an inhibitor of the Bruton’s tyrosine kinase) are being evaluated for the treatment of activated B-cell-like diffuse large B-cell lymphoma. To our knowledge, few such studies have been conducted to define the synergistic potential of other agents when combined with CDK4/6 inhibitors.

Numerous studies showed sensitivity of targeted therapy generated from patient-derived xenograft models that closely recapitulated the genetic and biological features in the original cancer patients [20]. PDX models that contain certain genetic feature like BRAF-mutated melanoma PDX models [21] or ER-positive breast cancer PDX models [22] have been selected as more clinically relevant models for targeted drug evaluation. Previously, we have established and conducted a population-based PDX trial consisting of 24 models established from 24 melanoma patients, which demonstrated robust antitumor effect of palbociclib in CDK4-amplified melanoma [23]. Thus, molecularly defined PDXs cohort study could serve as a preferred pipeline for targeted drug evaluation and translation, especially for the repurposing of FDA-approved agents.

Here, seeking to identify potential therapeutic agents to boost efficacy of CDK4/6 inhibition, we conducted a high-throughput combination drug screening to evaluate a collection of FDA-approved and investigational drugs against multiple molecularly defined HPV^neg^ HNSCC cell lines. After mapping the drug-drug interaction landscape using the screening data, we identified the most promising candidate combinations, which were further evaluated in vitro across an expanded panel of representative cell lines and in vivo using five molecularly annotated HPV^neg^ PDX models. Very briefly, our study provides preclinical evidence for particular targeted agents would synergize with palbociclib in HPV^neg^ HNSCC patients.

## Methods

### Cell cultures

HPV^neg^ cell lines used in this study were HN6 (RRID: CVCL_5516), HN30 (RRID: CVCL_5525), CAL27 (RRID: CVCL_1107), CAL-33 (RRID: CVCL_1108), SCC4 (RRID: CVCL_1684), SCC9 (RRID: CVCL_1685), PECA-PJ15 (RRID: CVCL_2678), PECA-PJ41 (RRID: CVCL_2680), FADU (RRID: CVCL_1218), UPCI-SCC-172 (RRID: CVCL_2231), UPCI-SCC-131 (RRID: CVCL_2229), Detroit-562 (RRID: CVCL_1171), and HSC-2 (RRID: CVCL_1287). Human breast cell line MCF-7 (RRID: CVCL_0031) was purchased from ATCC. All cell lines were routinely cultured at 37 °C with 5% CO_2_ according to the manufacturer recommendations. All cell lines were authenticated using short tandem repeat analysis and confirmed as mycoplasma-free (YEASEN). All cell lines were used for experiments within less than 20 passages.

### High-throughput drug screening

#### Chemical library

We purchased a chemical screening library composed of 162 compounds from Selleckchem for the combination drug screening with palbociclib. The corresponding information, including drug name, MoA (mechanism of action) and resource, is provided in Additional file 2: Table S1. The drugs used in cancer treatment and clinical trials were given priority, especially in HNSCC. To include different MoAs used in cancer treatment, our chemical library was constructed based on several common drug lists published in cancer studies [24, 25].

#### Combination high-throughput screening

In the primary drug combination screening, CAL27, HN6, FADU, and SCC9 cells were seeded into 96-well polystyrene tissue culture-treated Corning plates with 3000–6000 cells/well, depending on the specificity of each cell line. During the exponential growth phase, we added 10 μL of palbociclib and 10 μL 162 compounds (10X concentration) to individual wells for a 6×6 matrix screening (a 5-point custom concentration range, with constant 1:5 dilution between each point) (concentration ranging from 20 to 0.032 μM). Bortezomib (final concentration 3 μM) was used as a positive control for cell cytotoxicity. After 72 h of treatment, 10 μL of CCK8 (Beyotime) was added to each well. The plates were transferred to a standard incubator with a stainless steel lid for 2 h. OD values were taken using Synergy H1 (Biotek). All plates passed Quality Control conditions, with a Z factor greater than 0.6. In the secondary drug combination screening, cells were seeded into 384-well polystyrene tissue culture-treated plates at a concentration of 800–2000 cells/well. A total of 10 μL of compounds were dispensed for a 10×10 matrix screening (a 9-point custom concentration range, with constant 1:3 dilution between each point) (concentration ranging from 20 to 0.003 μM). The plates were incubated for 72 h in a standard incubator covered by a stainless steel gasketed lid to prevent evaporation. We added CCK8 and measured OD values. The efficacy of different drug combinations was estimated using the ExcessHSA score and a Bliss independence model.

### Cell viability assay

Cells were placed in 96-well plates to perform drug combination in three independent replicates. Cell viability was measured using Cell Counting Kit-8 assay according to the manufacturer’s instructions (Beyotime). To evaluate the effect of drug combination, cells were treated with each drug individually or in combination for 72 h. Data were processed in GraphPad Prism 5.0 (GraphPad Software, Inc.). Combination index (CI) was determined using CalcuSyn software (Biosoft), which allowed us to measure drug synergy using the median effect model (T.-C. Chou) [26]. A CI lower than 1.0 was considered to be synergistic.

### Cell-cycle analysis

After 48 h of palbociclib or combination treatment, HNSCC cells were harvested, washed, fixed in 75% ethanol overnight, and centrifuged at 300*g* for 5 min. The pellets were then washed and resuspended in 500 μL PI/RNase Staining Buffer (BD Pharmingen™). Cell-cycle analysis was performed on a BD Facscalibur. Data were processed using FlowJo v.10 (FlowJo LLC).

### EdU assay

Cell proliferation ability was measured using a Click-it EdU imaging kit (Beyotime Biotechnology, Alexa Flour 555). Briefly, post-treatment cells were incubated in culture medium with 10 μM EdU for 2 h. After fixation with 4% PFA and permeabilization with 0.3% Triton X-100, cells were incubated in Click-iT Plus reaction cocktail at room temperature for 30 min. Cell nuclei were stained with 10 μg/mL Hoechst 33342 for 2 h. Images were captured with Axio Vert.A1 and analyzed using ZEN software.

### Whole exome sequencing and data analysis

All HPV^neg^ cell lines, PDXs, and paired patients’ tumors were subjected to whole exome sequencing. DNA concentration of the enriched sequencing libraries was measured with the Qubit 2.0 fluorometer dsDNA HS Assay (Thermo Fisher Scientific). Size distribution of the resulting sequencing libraries was analyzed using Agilent BioAnalyzer 2100 (Agilent). DNA sequencing was performed with paired-end 2 × 150 base reads on the Illumina NovaSeq6000 platform at Mingma Technologies Co., Ltd. Raw FASTQ files were initially processed by a proprietary algorithm to filter out contaminated mouse sequencing reads.

#### Somatic mutations detection

For each paired sample, somatic SNVs and InDels were detected with Sentieon TNseq. We excluded mutations in low-complexity regions, including tandem repeats and highly homologous regions. Low-confidence variants were removed in case any of the following criteria was not satisfied: total depth > 10, alternative allele depth > 3 and mutation frequency > 0.01. All high-confidence mutations were then annotated with ANNOVA (Version 2016-02-01).

#### Copy number variant detection

We used CNVkit to analyze somatic copy number variations (CNVs). CNVs were called by comparing normalized tumor and normal data. Regions with absolute log2 copy number ratios at least 0.58 (= log2 (1.5)) were viewed as losses (deletions) or gains (amplifications) [27].

### RNA sequencing and data analysis

RNA sequencing was performed according to our previous research [28]. Briefly, cells were treated with either a drug or vehicle for 48 h, and then lysed in Trizol and stored at – 80 °C. Total RNA was extracted using the RNeasy Mini Kit (Qiagen) following the manufacturer’s instructions. We checked RIN number to evaluate RNA integrity using Agilent Bioanalyzer 2100 (Agilent technologies, Santa Clara, CA, US). Qualified total RNA was further purified using the RNAClean XP Kit (Beckman Coulter, Inc. Kraemer Boulevard Brea, CA, USA) and RNase-Free DNase Set (QIAGEN, GmBH, Germany). Library preparation was completed with the Illumina TruSeq RNA Sample Preparation Kit (Illumina). RNA sequencing was performed on a Illumina Hiseq 2000 platform. Raw reads were trimmed with Skewer (v0.2.2) to remove adapter sequences, and then aligned to the reference human genome GRCh37/hg19 with STAR (v2.4.2a). Gene expression abundance was estimated using RSEM (1.2.29) based on reads mapping uniquely to specific parts of the human genome. The presence of differentially expressed genes (DEGs) was determined using a negative binomial model with EdgeR, by comparing DMSO- and drug-treated tumors. *P*-values were calculated using the Wald test after controlling for multiple testing using B-H procedure. A |fold change| > 2 and an adjusted *P*-value < 0.05 were used as cut-off criteria.

Gene Set Enrichment Analysis (GSEA) was performed on normalized RNA-Seq expression data using the Desktop Application [29] and using the genes whose average CPM value was greater than 100 in at least one of the tested conditions (either DMSO or any of the 6 drug-treated sets). For each individual treatment vs. DMSO or palbociclib, a pre-ranked GSEA was performed based on the log2FC values, measured against “Hallmark” gene sets and epithelial or mesenchymal gene sets (Additional file 2: Table S2). Weighted enrichment statistics were based on 1000 gene-set permutations. Only the gene sets with an FDR adjusted *q*-value < 0.1 were selected for further analysis.

### Quantitative real-time PCR

Total RNA was extracted using TRIzol reagent (Invitrogen) following the manufacturer’s instructions. RNA was reverse transcribed using a Prime-Script RT Reagent Kit (Takara). Quantitative real-time PCR (qRT PCR) was conducted using a Roche LightCycler system with SYBR Green Reagent (Takara). Gene expression levels were normalized based on the GAPDH level. The primers used for qRT PCR are listed in Additional file 2: Table S3. Data were analyzed using the 2^−ΔΔCT^ method.

### Western blot analysis

Cells were lysed in SDS Lysis Buffer supplemented with protease and phosphatase inhibitor cocktail. Protein concentration was determined using BCA assay. Gel electrophoresis was performed on a stacking gel (90 V) and a separation gel (120 V). The protein samples were then transferred to PVDF membranes with 300 mA for 1.5 h. The membranes were incubated with primary antibodies at 4 °C overnight (Additional file 2: Table S4). Horseradish peroxidase (HRP) and secondary antibodies were used at room temperature for 1 h in the following day. Ultimate blots were visualized with enhanced chemiluminescence (ECL; Thermo Fisher Scientific).

### Generation of stable RRM2-overexpressing cell lines

pHBLV-CMV-MCS-3FLAG-EF1-ZsGreen-T2A-PURO and vector (Hanbio) were constructed and transfected into FADU and CAL27 cells using Fugene HD [30]. RRM2 overexpression was validated by Western blot analysis. Stable cells were routinely cultured and authenticated according to the ATCC guidelines.

### Histology and immunohistochemistry

Patient tissues and PDXs were embedded in paraffin. After this, we generated 4-μm-thick paraffin sections. Tissue morphology was determined by hematoxylin and eosin (H&E) staining. For immunohistochemistry (IHC) assays, paraffin sections were dewaxed and rehydrated through a graded ethanol series. After being treated with heat-mediated antigen retrieval using Citrate Unmasking Solution (Cell Signaling Technology), the sections were blocked with goat serum at room temperature for 1 h. Primary antibodies (Additional file 2: Table S4) diluted in 3% BSA were incubated at 4 °C overnight. We used DAB (Cell Signaling Technology) to perform the color reaction. All H&E and IHC images were obtained using an OLYMPUS microscope BX51 and a DP 71 camera (OLYMPUS). The positive grade was determined by the immuno-reactive score (IRS), which was according to the staining intensity (negative = 0, weak = 1, moderate = 2, strong = 3) and the percentage of positive tumor cells (0%=0, 1–10%=1, 11–50%=2, 51–80%=3, 81–100%=4). IRS ranging from 0 to 12 was calculated from the values of the staining intensity multiplied by the percentage of positive cells [31].

### Cell migration and invasion assays

Cell migration and invasion assays were performed using Transwell assay with uncoated polycarbonate transwell inserts (Millipore) for migration, or BioCoatTM transwell champers (Corning) for invasion. 10 ~ 12 × 10^4^ transfected cells were seeded in the upper chamber. After staining with crystal violet, positive cells were counted and analyzed under the microscope.

### In vivo experiments

To measure the antitumor efficacy of palbociclib monotherapy, we suspended CAL27, FADU, and HN6 cells in the mixture of PBS and Matrigel (1:1). Immuno-deficient athymic BALB/c-nu/nu female mice (6-week-old) were used in this experiment. A total of one million cells were injected subcutaneously into the left flank region of each mouse. When tumor size reached at least 100 mm^3^, the mice were randomly divided into palbociclib and vehicle groups (6 mice in each group). Palbociclib (60 mg/kg) and vehicle (sodium lactate buffer, 50 mmol/L, pH 4.0) were orally administrated once a day. HN6 and FADU xenografts were treated with a continuous application of palbociclib for a total of 14 days, while CAL27 xenografts were treated with a phased schedule (days 1–14: palbociclib treatment; day 15–31: treatment suspension; day 32–69: palbociclib treatment).

In the drug combination experiment on FADU xenografts, the mice were randomly assigned into four groups receiving vehicle (sodium lactate buffer, 50 mmol/L, pH 4.0, orally, daily), palbociclib (60 mg/kg, orally, daily), alpelisib (20 mg/kg, orally, daily), or both for a total of 21 days. To further evaluate the efficacy of the clinical treatment, PDX models were used for different drug combinations. Xenograft-bearing mice were randomly assigned into seven groups (7 mice per group) to receive vehicle, palbociclib, alpelisib, cetuximab (1 mg/kg, intraperitoneal, twice a week), palbociclib plus cetuximab, palbociclib plus alpelisib, and palbociclib plus cisplatin (3 mg/kg, intraperitoneal, once a week). Treatment began when tumors reached at least 100 mm^3^. The treatment scheme for each model varied from 8 to 29 days (mice were sacrificed either when  tumor volume exceeded 1500 mm^3^ or at the experiment endpoint). Tumor size and bodyweight were measured twice per week. The percentage of tumor growth inhibition [32] was defined as 100 × [1 − (TVf_treated − TVi_treated)/ (TVf_control − TVi_control)]. TVi and TVf represent the mean tumor volume at the start and end of treatment, respectively [23].

### Statistical analysis

Statistical analysis was performed using GraphPad Prism 9.0 software. Data are presented as the mean ± S.D. or S.E.M., as per indicated in the figure legends. Pairwise comparisons between experimental and control groups were performed using unpaired two-tailed Student’s *t* tests, one-way ANOVA, or two-way ANOVA where appropriate. *P* < 0.05 was considered to be statistically significant. *P*-value symbols are denoted as follows: * (*P* < 0.05), ** (*P* < 0.01), *** (*P* < 0.001), and **** (*P* < 0.0001).

## Results

### Palbociclib monotherapy shows limited drug efficacy in HPV^neg^ HNSCC

**Fig. 1:**
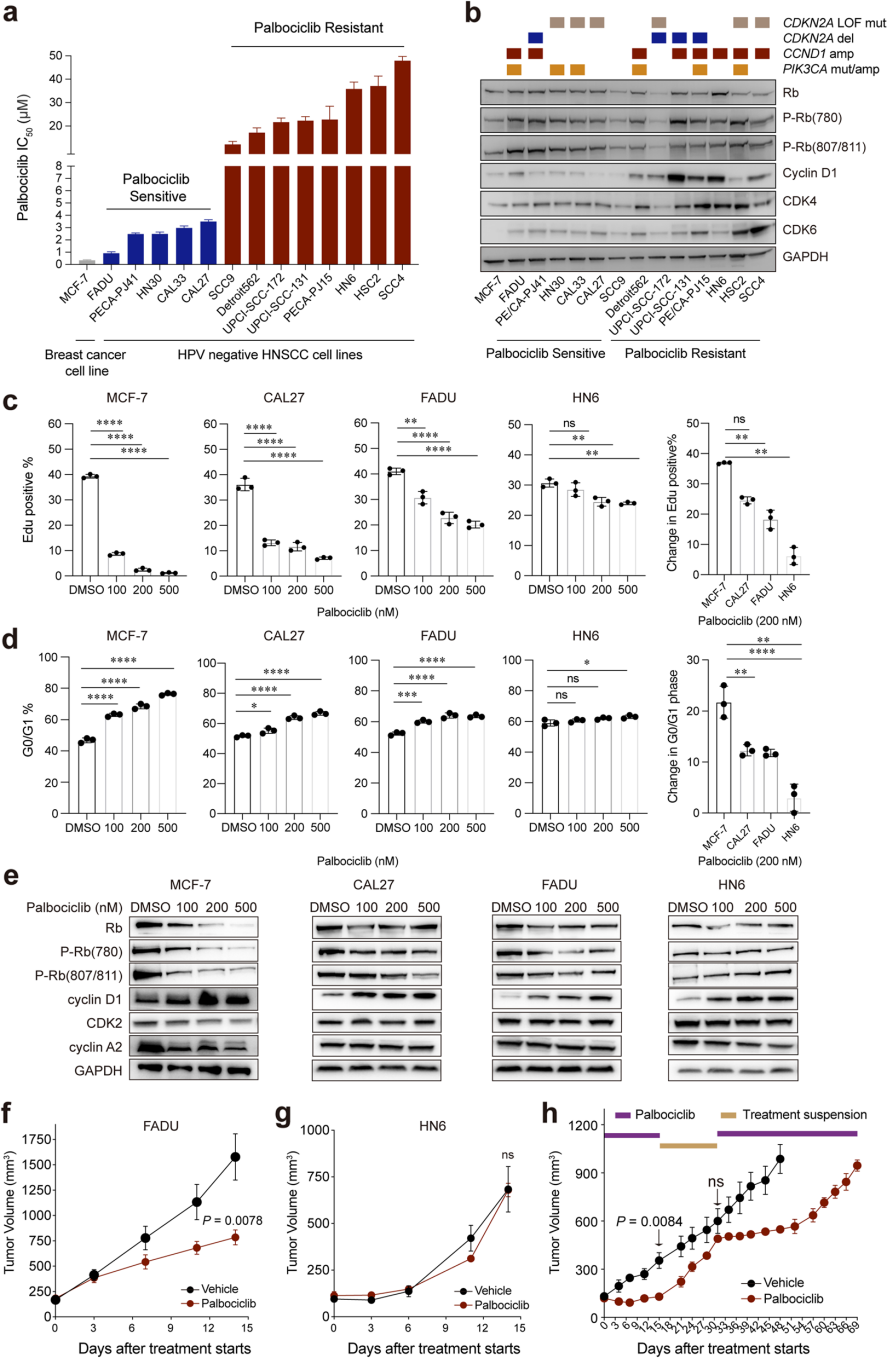
Limited response to CDK4/6 inhibitor palbociclib monotherapy in HNSCC cell lines and xenografts. **a** Palbociclib drug sensitivity identified by half-maximal inhibitory concentration (IC50) values (μM). Cell lines are colored as follows: grey—estrogen receptor-positive breast cancer; dark blue—palbociclib-sensitive HNSCC cell lines; dark red—palbociclib-resistant HNSCC cell lines. The data are shown as the mean ± SD. **b** Specific genetic alterations and Western blot analysis of genes involved in the cell-cycle pathway in 13 HNSCC cell lines and breast cancer cell line MCF-7. **c** Cell proliferation measured by EdU analysis after treatment with palbociclib for 48 h. The data are shown as the mean ± SD. ns, not significant. ** *P* < 0.01, **** *P* < 0.0001, as estimated by one-way ANOVA. **d** Flow cytometry analysis of HNSCC and MCF-7 cell lines treated with palbociclib for 48 h. We quantified the population of cells at the G0/G1 phase of the cell cycle. The data are shown as the mean ± SD. ns, not significant. * *P* < 0.05, ** *P* < 0.01, *** *P* < 0.001, and **** *P* < 0.0001, as estimated by one-way ANOVA. **e** Western blot analysis of cell-cycle-related proteins after treatment with palbociclib for 48 h. **f** Growth curve of FADU xenograft tumors (*n* = 8) treated with palbociclib for 14 days. **g** Growth curve of HN6 xenograft tumors (*n* = 6) treated with palbociclib for 14 days. **h** Growth curve of CAL27 (*n* = 5) xenograft tumors treated with a phased treatment schedule. Palbociclib treatment was conducted for 14 days (from day 1 to day 14, marked in purple line) and subsequently suspension (from day 15 to day 31, marked in yellow line) and retreatment (from day 32 to day 69, marked in purple line). The data are shown as the mean ± SEM. ns, not significant. *P*-values were estimated by two-way ANOVA

To evaluate the therapeutic efficacy of palbociclib monotherapy in HPV^neg^ HNSCC, we investigated the cellular response of CDK4/6 inhibition in different HPV^neg^ cell lines. An ER-positive MCF-7 human breast cancer cell, which has been demonstrated to be highly sensitive to palbociclib [33], was used as positive control. All of the 13 selected HPV^neg^ HNSCC cell lines showed varied degree of sensitivity to palbociclib, with a mean IC_50_ ranging from 0.908 to 47.88 μM, and we defined 5 cell lines with relative lower IC_50_ (< 5 μM) as “palbociclib sensitive” (PS) and the remaining 8 cell lines as “palbociclib resistant” (PR; Fig. 1a and Additional file 1: Fig. S1a, b). Well-recognized genetic lesions (*CDKN2A*, *CCND1* and *PIK3CA*) of all the 13 cell lines were annotated by whole exome sequencing (WES; Fig. 1b). Notably, the mutations identified in 7 of these 13 cell lines were similar to that which have also been characterized by Cancer Cell Line Encyclopedia [34] and Genomics of Drug Sensitivity in Cancer [25] database (Additional file 1: Fig. S1b and Additional file 2: Table S5). Additionally, the mRNA and protein expression levels of representative cell-cycle pathway-related genes were determined by qRT PCR and Western blot (Fig. 1b and Additional file 1: Fig. S1e).

Rb/pRb, whose expression serves as an inclusion eligibility criterion in several clinical trials of palbociclib [35, 36], was universally expressed among all the PS cell lines (Fig. 1b and Additional file 1: Fig. S1e). As expected, we found that significantly lower expressions of Rb1 mRNA, total Rb, and phosphorylated Rb at Ser807/811 protein were detected in PR group when compared with PS group (*P* = 0.0246, 0.0036, 0.0404, respectively, Additional file 1: Fig. S1c, e). On the other hand, the mRNA and protein expression levels of CDK6, which have been reported to be correlated with intrinsic resistance to palbociclib [37], were significantly higher in PR group when compared with PS group (*P* = 0.0347 and 0.0454, respectively, Additional file 1: Fig. S1c, e). However, *CDKN2A* deletion/mutation, *CCND1* or *CDK6* amplification, which contribute to Rb pathway activation and have been reported as predictive biomarkers of CDK4/6 inhibition [7, 38], were not found to be correlated with palbociclib sensitivity (Additional file 1: Fig. S1d), possibly due to insufficient sample size.

Two PS cell lines (PS-CAL27, PS-FADU), one PR cell lines (PR-HN6) and MCF7 were selected for evaluation of the cellular response to palbociclib treatment. When treated with different doses of palbociclib, PS-CAL27, PS-FADU and PR-HN6 showed significantly less reduction in DNA replication and G1 phase arrest when compared with MCF7 cells which displayed prominently decreased proliferation and G1-arrest as previously reported [39] (Fig. 1c, d and Additional file 1: Fig. S1f). Western blot analysis also showed palbociclib (ranging from 100 to 500 nM) exhibited limited inhibition of total and phosphorylated Rb (Rb and pRb), as well as the key regulators of G1/S-phase transition (CDK2 and Cyclin A2) (Fig. 1e).

Additionally, palbociclib monotherapy exerted significant inhibitory effects on the growth of PS-FADU xenografts in a 2-week treatment schedule, while it had no effects on PR-HN6 xenografts (Fig. 1f, g, Additional file 1: Fig. S1g). To evaluate the long-term therapeutic efficacy of palbociclib [40], an “on-off-on” phased treatment schedule was conducted on CAL27 xenografts (Additional file 1: Fig. S1h). As illustrated in Fig. 1h, palbociclib monotherapy exerted significantly inhibitory effects on the growth of PS-CAL27 xenografts after the first treatment phase (days 1–14, *P* = 0.0084). During the treatment suspension phase, tumor progressed rapidly upon the removal of palbociclib (days 15-31, treatment off), reflecting the cytostatic rather than cytotoxic effect of CDK4/6 inhibition. When the mean tumor volumes in the treatment group reached comparable to that of the control group, palbociclib was resumed and PS-CAL27 xenografts were first responsive to palbociclib (from days 31–52) while started to regrow and progressed under drug pressure (from day 53 to day 69), indicating the emergence of acquired resistance to palbociclib in PS-CAL27 (Fig. 1h and Additional file 1: Fig. S1g). Taken together, HPV^neg^ HNSCCs tend to be less responsive or develop drug resistance to treatment with palbociclib monotherapy, highlighting the need to investigate combinational therapeutic strategies.

### Quantitative high-throughput 6 × 6 matrix combination screening identifies synergistic agents

**Fig. 2:**
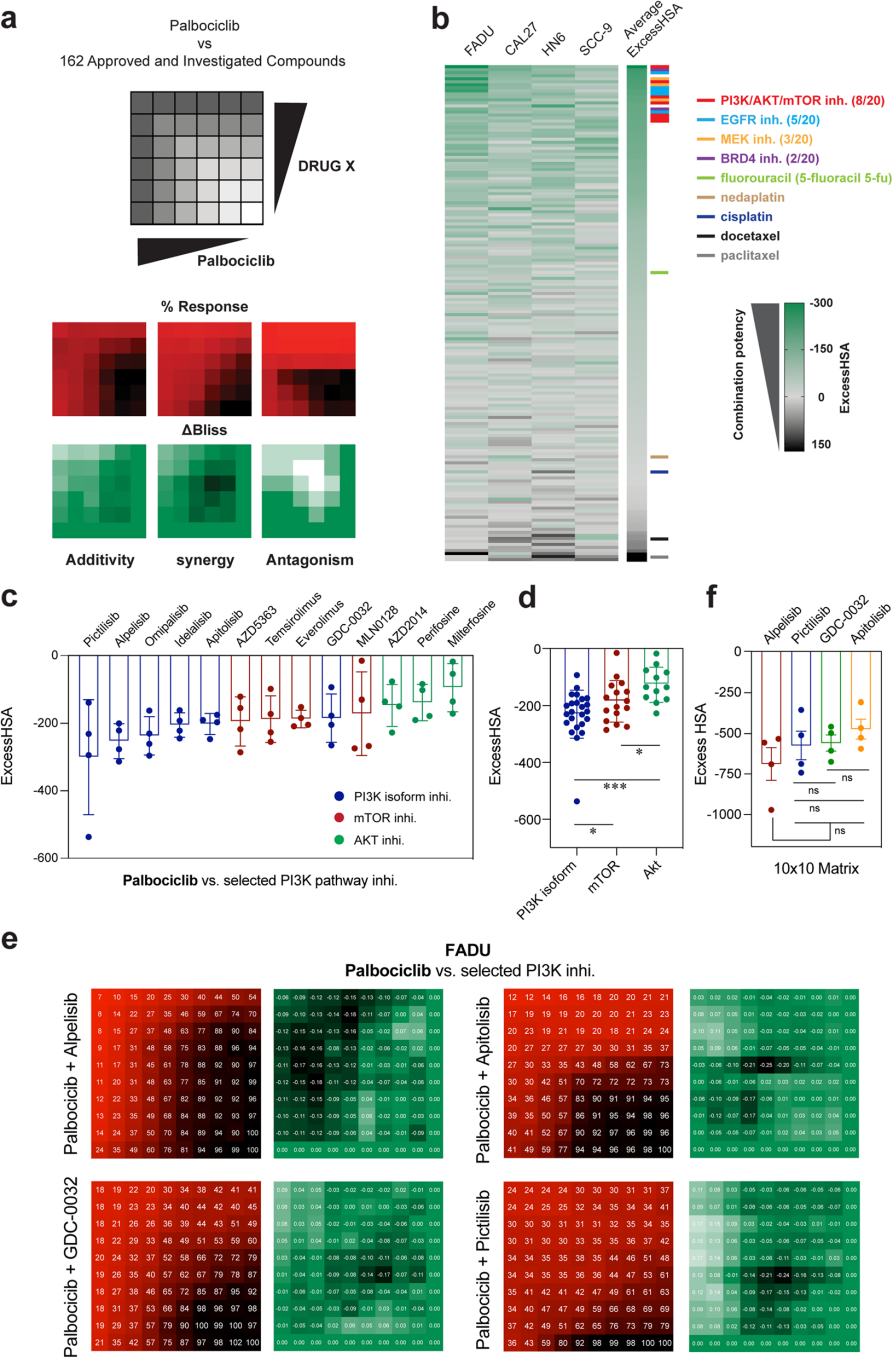
High-throughput combination drug screening identified palbociclib-based drug-drug interactions in HNSCC. **a** Schematic layout of the palbociclib screening versus 162 different compounds. Top: each drug pair was tested in a 6 × 6 matrix block, with five doses plus DMSO control. Bottom: examples of percent response and ∆Bliss heat maps for additivity, synergy, or antagonism outcomes. **b** The hierarchal view of drug screening was ranked by synergy, as assessed by the average ExcessHSA metric. Top colorful bars highlight drugs from key mechanistic classes, including PI3K/AKT/mTOR (red), EGFR (blue), MEK (orange), BRD4 (purple) inhibitors, fluorouracil (light green), nedaplatin (light brown), cisplatin (dark blue), docetaxel (black), and paclitaxel (grey). The numbers in brackets represent the number of inhibitors in top 20 synergistic compounds. **c** Individual ExcessHSA values of palbociclib combined with 13 PI3K pathway inhibitors from the screening library that were classified as PI3K isoform (blue), AKT (green), and mTOR (red) inhibitors. **d** Bar plot showing the ExcessHSA in different kinds of PI3K pathway. Data are shown as the mean ± SD. *P < 0.05 and *** *P* < 0.001, as estimated by two-way ANOVA. **e** The 10×10 heat maps of % Response and ∆ Bliss palbociclib combinations with pictilisib, alpelisib, taselisib (GDC-0032), or apitolisib in FADU. **f** The ExcessHSA values for palbociclib combination with the four PI3K inhibitors shown in **e**. Data are shown as the mean ± SD. ns, not significant, as estimated by two-way ANOVA

To identify alternative combinational approaches for palbociclib in HPV^neg^ HNSCC, we performed high-throughput combination drug screening in four HNNCC cell lines with varied sensitivity to palbociclib (PS-CAL27, PS-FADU, PR-SCC9, PR-HN6) using a customized library within 6 × 6 checkerboard matrix (Fig. 2a). The library includes 162 agents covering multiple inhibitors for well-explored oncogenic targets [for instance, phosphoinositide 3-kinase (PI3K), anaplastic lymphoma kinase (ALK), and mitogen-activated protein kinase (MEK)] and targeting 54 distinct mechanisms of action (Additional file 2: Table S1). Notably, most of the FDA-approved agents that are currently used in HNSCC treatment including chemotherapeutics (cisplatin, docetaxel, 5-fluorocrail) and targeted therapy (cetuximab) were also incorporated in the library [41].

The 6 × 6 discovery checkerboard matrix generated a total of 16,200 drug-drug interactions. Of the 648 discrete screened plates, the average *Z*-prime score as a control for robustness was 0.83, and *Z*-prime score of all plates was greater than 0.5, indicating that the screening assay was experimentally robust (Additional file 1: Fig. S2a, b). Each 6×6 matrix was scored by the sum of ExcessHSA (Excess over Highest Single Agent) for evidence of synergistic (ExcessHSA score < − 20), additive (− 20 ≤ ExcessHSA score ≤ 20), or antagonistic effects (ExcessHSA score > 20, Additional file 2: Table S6), and the average ExcessHSA score of each compound in four cell lines was ranked accordingly (Fig. 2b).

Overall, we found that 77.16% of these compounds (125/162) showed distinct patterns of synergistic effect with palbociclib and ranked all compounds based on average ExcessHSA scores (Fig. 2b). The most obvious trend was that 7 PI3K pathway inhibitors (e.g., pictilisib, everolimus, alpelisib) were among the top 20 hits for synergism with palbociclib. It was also notable that receptor tyrosine kinase (RTK) related agents including 5 EGFR inhibitors or antibody (e.g., osimertinib, dacomitinib, and cetuximab) and 3 MEK inhibitors (e.g., binimetinib, cobimetinib) were ranked among the top 20 hits in the combination drug screening. Bromodomain and extra-terminal protein (BRD4) has been increasingly appreciated as a key oncogene during the tumorigenesis and development of HNSCC [42]. Two BRD4 inhibitors, JQ1 and GSK-525762A, demonstrated significant synergy with palbociclib (ranking the 2nd and 18th). On the other hand, we found that most of the evaluated conventional chemotherapies displayed additive or antagonistic effects when combined with palbociclib (e.g., cisplatin, nedaplatin, ExcessHSA -7.63, -13.85; paclitaxel, docetaxel; ExcessHSA 173.86, 62.76, Fig. 2b). Taken together, the quantitative high-throughput matrix combination screening characterized the synergistic potential of a list of well-recognized agents when combined with palbociclib.

### Quantitative 10 × 10 matrix screening validates PI3K inhibitors as top synergistic option

Considering the significant synergistic effect observed between palbociclib and the PI3K pathway inhibitors, we conducted a more detailed analysis of 13 agents targeting different components of PI3K pathway, including 6 PI3K inhibitors, 3 AKT inhibitors, and 4 mTOR inhibitors. Four of the top 7 PI3K pathway inhibitors belongs to isoform-selective PI3K inhibitors, including PI3Kα/δ inhibitor pictilisib (ExcessHSA: − 300.41), FDA-approved PI3Kα selective inhibitor alpelisib (ExcessHSA: − 227.83), dual PI3K/ mammalian target of rapamycin (mTOR) inhibitors apitolisib (ExcessHSA: − 202.16), and PI3Kα/β/γ inhibitor taselisib (GDC-0032) (ExcessHSA: − 185.12, Fig. 2c). Three AKT inhibitors (e.g., AZD5363, perifosine, miltefosine) and 4 mTOR inhibitors (e.g., temsirolimus, everolimus, MLN0128, AZD2014) also showed strong synergistic effects with palbociclib (average ExcessHSA: − 194.86, − 138.83, − 94.83, and − 188.08, − 187.27, − 171.81, − 147.45, respectively, Fig. 2c). Notably, PI3K inhibitors showed significantly higher average ExcessHSA when compared with AKT inhibitors and mTOR inhibitors (− 230.47 versus − 127.04 and − 185.5, *P* = 0.0002 and 0.039, respectively, Fig. 2d).

To capture more comprehensive information about drug-drug interactions, we conducted a 10 × 10 matrix study to evaluate the synergistic scores by incorporating up to 81 different concentration combinations. As defined by multiple metrics including the ExcessHSA score and Bliss independence model, this experiment further confirmed the synergistic effects between palbociclib and the four PI3Ki (ranging from − 476.86 to − 690.54), and alpelisib demonstrated the most synergistic effect when combined with palbociclib (Fig. 2e, f; Additional file 1: Fig. S2c). Moreover, we found that *PIK3CA*-amplified cell line FADU outperformed other cell lines with the highest average ExcessHSA score of these combinations (− 748.28, Additional file 1: Fig. S2d). Thus, these screening results indicated that PI3K pathway contributed the most to sustain the viability of HNSCC under palbociclib treatment, with PI3K inhibitors being identified as the most promising combination option.

### PI3K inhibitors show enhanced synergistic effect in PIK3CA-altered HPV^neg^ HNSCC

**Fig. 3:**
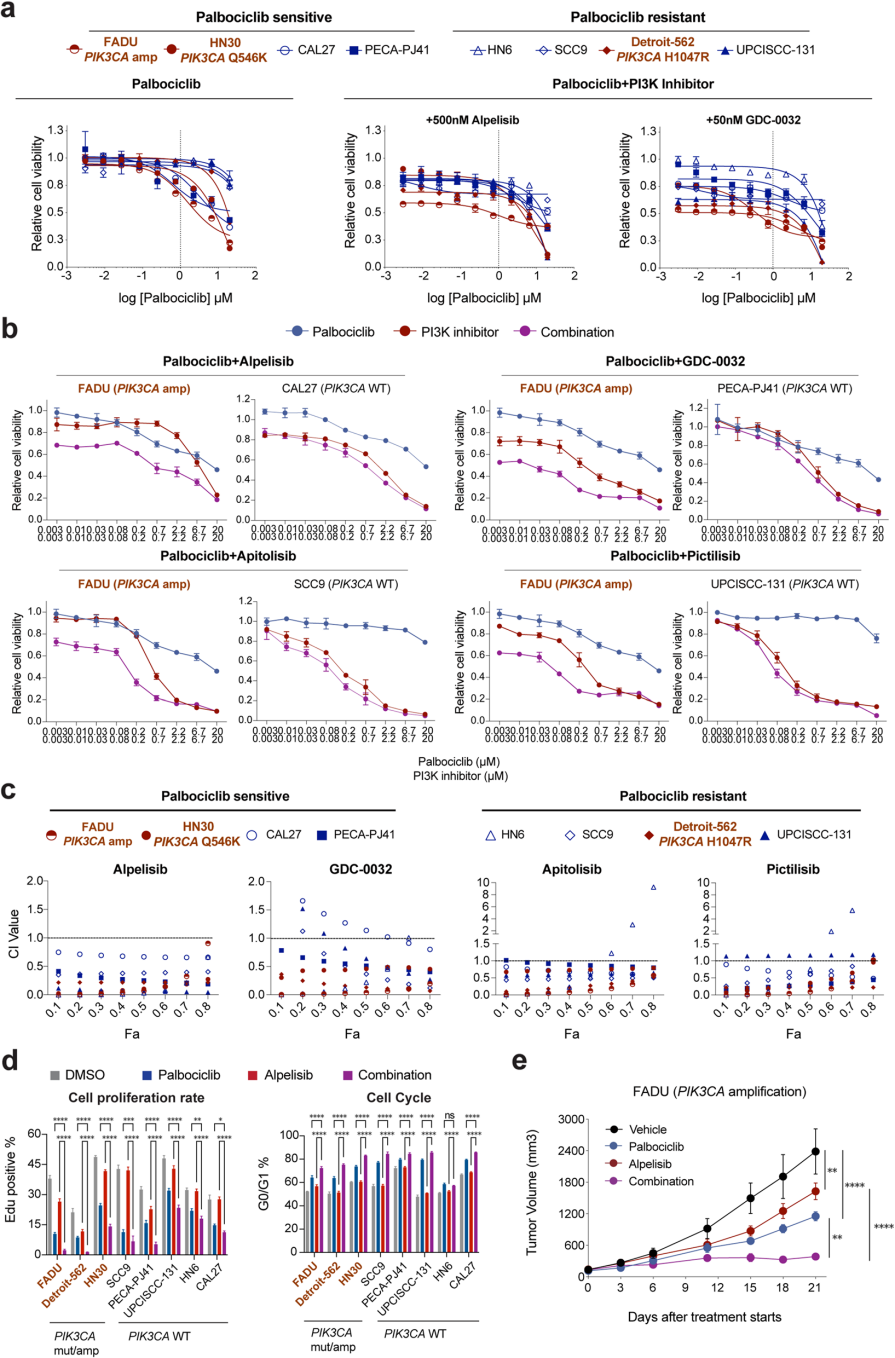
In vitro and in vivo validation of drug combinations in HNSCC cell lines and xenografts. **a** Dose-response curves of cell viability after palbociclib monotherapy and palbociclib combined therapy with 500 nM alpelisib and 50 nM GDC-0032 for 72 h in palbociclib sensitive and resistant HNSCC cell lines (red, PIK3CA mut/amp; blue, PIK3CA WT). **b** Dose-response curves of cell viability at gradient concentrations of palbociclib (blue), PI3K inhibitors (red), or combined Palbociclib-PI3K (purple) therapies for 72 h in representative PIK3CA mut/amp and PIK3CA WT HNSCC cell lines. See the remaining cell lines in each combination in Additional file 1: Fig. S3b-e. **c** Fa-CI dot plots show the CI values used to identify drug synergies between palbociclib and four PI3K inhibitors in all 8 experimentally examined HNSCC cell lines from each combination (red, PIK3CA mut/amp; blue, PIK3CA WT). Horizontal dashed line indicates CI = 1. Values below this threshold indicate drug synergy, while those above it represent drug antagonism. Fa (fraction affected), CI (combination index). **d** Cell proliferation measured by EdU analysis after treatment with DMSO vehicle (control; gray), palbociclib (blue), alpelisib (red), and palbociclib-alpelisib combination (purple) for 48 h (left). Population of cells at the G0/G1 phase of cell cycle estimated by flow cytometry analysis after the treatments specified above (right). The data are shown as the mean ± SD. * *P* < 0.05, ** *P* < 0.01, *** *P* < 0.001, and **** *P* < 0.0001, as estimated by two-way ANOVA. **e** Tumor growth of FADU cell xenograft tumors after treatment with vehicle, palbociclib, alpelisib, and palbociclib-alpelisib combination (*n* = 5). The data are shown as the mean ± SEM. ** *P* < 0.01, **** *P* < 0.0001, as estimated by two-way ANOVA. mut/amp, mutation or amplification; WT, wild type

*PIK3CA* is the most commonly altered oncogene in HNSCC, with mutations or amplifications detected in 34% of all the HPV-negative HNSCC tumors and previous studies have reported that *PIK3CA* mutation or amplification was predictive for the response of PI3K/AKT/mTOR inhibitors [43]. To assess whether PI3K inhibitors plus palbociclib would lead to more prominent combination potency in *PIK3CA-*altered cell lines, we then evaluated these inhibitors (alpelisib, pictilisib, GDC-0032, and apitolisib) in combination with palbociclib in eight selected cell lines (4 PS and 4 PR), which represent two major genetic subtypes (*PIK3CA* mut/amp: Detroit-562, FADU and HN30; *PIK3CA* wild type (WT): HN6, PECA-PJ41, SCC9, CAL27, and UPCI-SCC-131). Drug dose-response curves showed that all four PI3K inhibitors resulted in obvious shifts of drug response curve and corresponding decreased IC_50_ when combined with palbociclib, especially for three *PIK3CA-*altered cell lines (Fig. 3a; Additional file 1: Fig. S3a).

We next measured cell viability across different drug doses or combinations in each cell line. The result showed that combinational treatment with PI3K inhibitors resulted in stronger anti-proliferation activities in *PIK3CA* mut/amp cell lines when compared with others (*PIK3CA* WT) at low concentrations (Fig. 3b; Additional file 1: Fig. S3b-e). The resulting combination index (CI) theorem of Chou-Talalay offers quantitative definition for additive effects (CI = 1), synergism (CI < 1), and antagonism (CI > 1) in drug combinations [26]. Analysis with this algorithm indicated strong synergy between palbociclib plus alpelisib, and to a lesser degree, palbociclib plus GDC-0032, palbociclib plus apitolisib, and palbociclib plus pictilisib (Fig. 3c). Importantly, the highest extent of synergism was observed in three *PIK3CA* mut/amp cell lines, suggesting that disruption of PI3K pathway substantially sensitized *PIK3CA* mut/amp HNSCC cells to palbociclib-mediated cytostatic effect (Fig. 3c). Notably, significant synergetic effect of these combinations was also observed using another CDK4/6 inhibitor, abemaciclib, with four PI3K inhibitors, especially in *PIK3CA* mut/amp cell lines (Additional file 1: Fig. S4). Additionally, combinations of palbociclib and PI3K inhibitors showed consistently synergistic effects on cell proliferation and G0/G1 cell cycle arrest across all 3 *PIK3CA* mut/amp cell lines while divergent synergistic effects were observed in 5 *PIK3CA* WT (Fig. 3d and Additional file 1: Fig. S3f-h). Alpelisib, which showed the most consistently synergistic effect in almost all cell lines, was further evaluated as a combinational agent with palbociclib in vivo using FADU-derived xenograft (annotated with *PIK3CA* amplification). As expected, palbociclib or alpelisib monotherapy slightly delayed tumor progression, while palbociclib plus alpelisib showed significantly synergistic therapeutic efficacy (Fig. 3e and Additional file 1: Fig. S3i-k). Thus, we found that alpelisib had a broad synergistic effect in HPV^neg^ HNSCC cell lines, with particularly higher therapeutic potential in cases harboring *PIK3CA* alterations.

### Alpelisib exerts synergistic effect via suppression of EMT induced by palbociclib

**Fig. 4:**
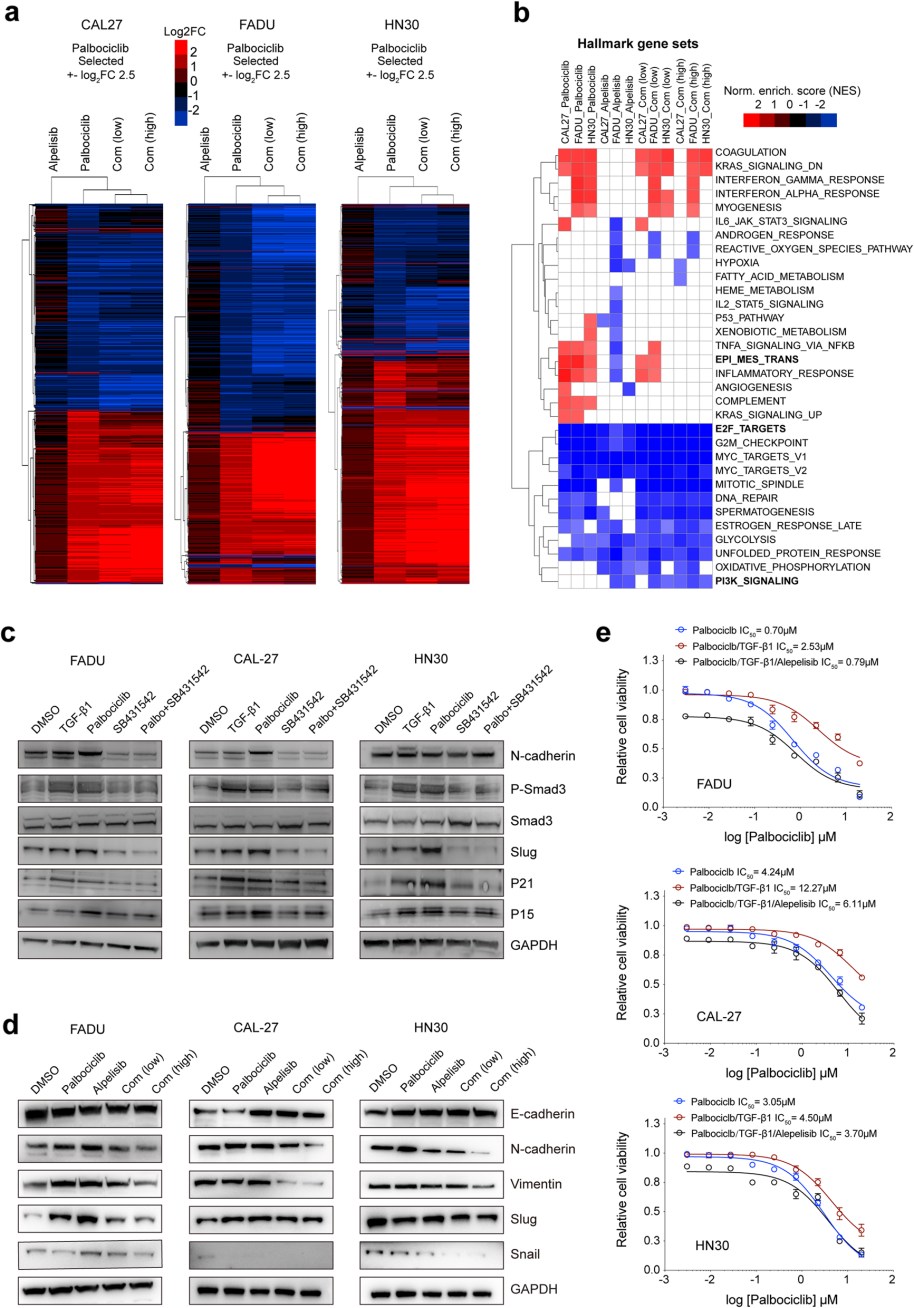
Alpelisib exerts synergistic effect via suppression of EMT induced by palbociclib. **a** Heat map showing an unsupervised hierarchical clustering of gene expression after treatment with 1 μM palbociclib, 1 μM alpelisib, a low dosage of combined palbociclib alpelisib (500 nM of each drug), and a high dosage of this combination (1 μM of each drug) in CAL27, FADU, and HN30 cell lines. **b** Hallmark gene sets are listed. E2F targets genes, EPI_MES_TRANS, and PI3K signaling are marked in bold. **c** Western blot analyses of EMT markers, TGF-β1/Smad3, and downstream genes were performed in three HNSCC cell lines incubated for 72 h with vehicle (DMSO), 10 ng/ml TGF β1, 1 μmol/L palbociclib, 2 μmol/L SB-431542, or both inhibitors. **d** Western blot analysis of EMT markers were performed in three HNSCC cell lines incubated for 72 h with vehicle (DMSO), 500 nmol/L palbociclib, 500 nmol/L alpelisib, a low dosage of combination (500 nM of each drug), or a high dosage of combination (1 μM of each drug). **e** Dose-response curves of relative cell viability with palbociclib monotherapy, palbociclib monotherapy supplemented with 10 ng/ml TGF-β1, and palbociclib monotherapy supplemented with 10 ng/ml TGF-β1 and 500 nM alpelisib for 72 h in three HNSCC cell lines

To explore potentially synergistic mechanisms between palbociclib and alpelisib, we performed RNA-seq analysis on PS-FADU, PS-HN30, and PS-CAL27 cells treated with DMSO, palbociclib, alpelisib, or two combination treatments using low or high doses of alpelisib plus palbociclib. Significantly upregulated and downregulated genes were then identified in palbociclib, alpelisib, and combination treatment groups when by comparison with the DMSO group (Fig. 4a). As expected, gene set enrichment analysis (GSEA) analysis showed cell-cycle-related genes (including E2F targets, G2M, and MYC target-related genes) were consistently downregulated in all treatment groups, while PI3K signaling pathway genes were only downregulated by alpelisib and the combination treatments (Fig. 4b). Leading-edge analysis from GSEA gene sets indicated that E2F targets and PI3K signaling pathway genes were both strongly downregulated in the treatment groups of all three cell lines (Additional file 1: Fig. S5a, b). Western blot analysis of the combination treatment group in all three cell lines showed decreased levels of E2F targets (e.g., Cyclin A2 and Aurora B) and PI3K pathway markers (e.g., pAKT and pS6 phosphorylation, Additional file 1: Fig. S5d).

Epithelial mesenchymal transition (EMT) has been reported to be associated with resistance to targeted therapy through various mechanisms of action [44, 45]. More recently, a study demonstrated that HPV^neg^ HNSCC cancer cells harboring a mesenchymal phenotype were less sensitive to CDK4/6 inhibition than those with an epithelial phenotype [8]. In agreement with these reports, we found that genes related to EMT were consistently upregulated under palbociclib treatment in all three cell lines (Fig. 4b). Previous study has demonstrated that CDK4/6 knockdown could upregulate TGF-β/Smad3 signaling and induce EMT via increasing the expression of downstream targets including p15 and p21 [46–48]. To investigate whether palbociclib treatment elicited EMT through TGF-β/Smad3 signaling in HNSCC, CAL27, FADU, and HN30 cells were next incubated with TGF-β1, SB-431542 (a TGF-β type I receptor kinase inhibitor) and palbociclib in the absence or presence of SB-431542. We first observed that TGF-β1 activated TGF-β/Smad3 signaling as evidenced by increased phosphorylated Smad3 and Smad3 downstream targets p15/p21, which were similarly upregulated under palbociclib treatment (Fig. 4c). Notably, SB-431542 completely inhibited the expression of palbociclib- or TGF-β1 (a well-known EMT inducer)-mediated induction of p15/p21 and EMT-associated genes, including N-cadherin and Slug (Fig. 4c). Thus, these results indicated that the EMT elicited by CDK4/6 inhibition was largely caused by TGF-β/Smad3 signaling-mediated induction of p15 and p21.

As for the combination treatment groups, we observed that low-dose combination resulted in a lower degree of EMT gene-set upregulation compared to that under palbociclib monotherapy, while no upregulation of the EMT gene set was detected in high-dose combination treatment group of any of the three cell lines (Fig. 4b). Leading-edge analysis from the GSEA EMT gene set highlighted genes that were strongly up- and downregulated only in combination-treated cells (Additional file 1: Fig. S5c). Western blot analysis showed that the combination treatments resulted in decreased levels of representative mesenchymal proteins including N-cadherin, Vimentin, and Snail whereas epithelial protein E-cadherin appeared to be unaffected in all three cell lines under the combination treatments (Fig. 4d). Moreover, cell viability assay showed that TGF-β1 treatment effectively increased the palbociclib resistance (a 1~4-fold increase in IC_50_ values of palbociclib) in all three HNSCC cell lines, and PI3K inhibitor alpelisib combined with palbociclib could at least partially restore palbociclib sensitivity (Fig. 4e). Collectively, these results show palbociclib plus alpelisib does not induce the EMT and alpelisib might exert synergistic effect through suppressing EMT-related impacts induced by palbociclib.

### Evaluation of selected palbociclib-based treatment combinations in vitro

**Fig. 5:**
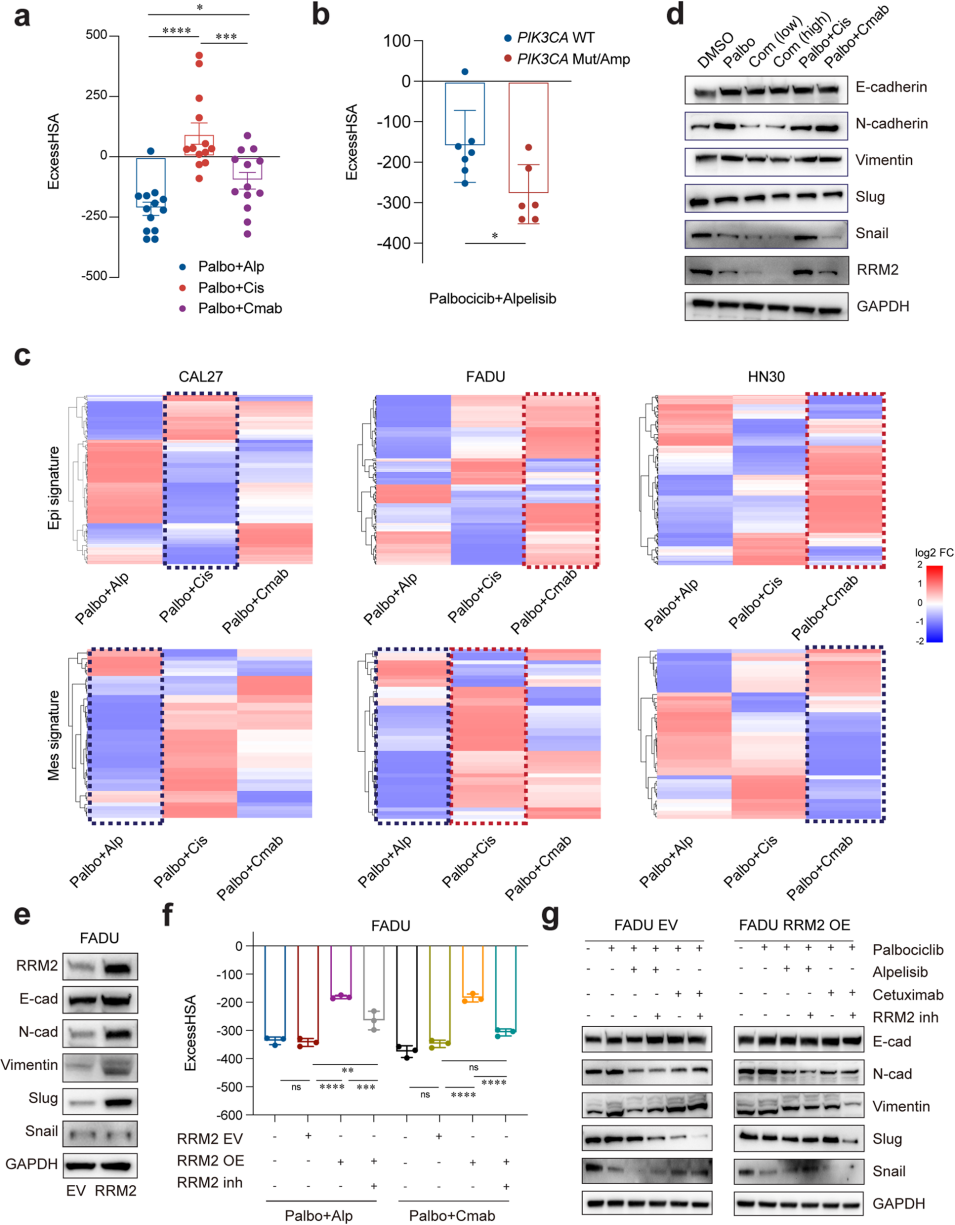
EMT signaling mechanistic analysis of selected drug combinations in HNSCC in vitro. **a** ExcessHSA scores of palbociclib-alpelisib, palbociclib-cetuximab, and palbociclib cisplatin combined therapy in 13 HNSCC cell lines mentioned in Fig. 1a. **b** ExcessHSA scores of palbociclib-alpelisib combined therapy in PIK3CA WT and mut/amp cell lines. **c** Gene expression of epithelial and mesenchymal signatures after three distinct treatments (palbociclib plus alpelisib, palbociclib plus cetuximab, palbociclib plus cisplatin) compared to palbociclib monotherapy in HNSCC cell lines. Significantly up- and downregulated genes were marked with red and blue dotted lines, respectively. **d** Protein expressions of canonical EMT markers in FADU cells treated with palbociclib, palbociclib plus alpelisib (low dosage), palbociclib plus alpelisib (high dosage), palbociclib plus cisplatin, and palbociclib plus cetuximab. **e** RRM2 and canonical EMT marker expressions in RRM2-overexpressing FADU cells. **f** ExcessHSA scores were determined in order to observe the effects of palbociclib combined with alpelisib or cetuximab for 72 h in the presence of RRM2 overexpression and the corresponding inhibitor osalmid. **g** Canonical EMT markers expressions were detected in both EV- and RRM2-expressing FADU cells after palbociclib monotherapy and combination treatments in the presence/absence of the RRM2 inhibitor. The data are shown as the mean ± SD. n.s., not significant; * *P* < 0.05, *** *P* < 0.001, **** *P* < 0.0001, as estimated using unpaired two-tailed Student’s *t* tests, one- or two-way ANOVA analysis

Cisplatin is a standard therapeutic regimen for HNSCC [49], while the EGFR-targeted antibody cetuximab, except for anti-PD-1 antibody, remains the only molecular therapy approved for the treatment of HNSCC [50]. Thus, various treatment strategies using these two agents as combinational agents are currently under active clinical investigation in HNSCC [51]. To this end, the therapeutic efficacy of these three combinations: palbociclib plus alpelisib, along with palbociclib plus cetuximab and palbociclib plus cisplatin, were further examined in a total of 13 HPV^neg^ cell lines. We found that palbociclib plus alpelisib led to the highest degree of synergism in inhibiting tumor cell growth of the three combinations (average ExcessHSA score: − 213.24; Fig. 5a), with significantly higher ExcessHSA scores observed in *PIK3CA* mut/amp cell lines than that of *PIK3CA* WT lines (*P* = 0.0253, Fig. 5b). Palbociclib plus cetuximab also exhibited synergistic effects, but to a lesser extent (average ExcessHSA score: − 99.33). By contrast, palbociclib plus cisplatin combination resulted in antagonistic effects in 8 of the 13 examined cell lines (average ExcessHSA score: 96.12; Fig. 5a and Additional file 1: Fig. S6a).

To further evaluate whether EMT was involved in these synergistic or antagonistic effects, RNA-seq was conducted in PS-FADU, PS-HN30, and PS-CAL27 cells treated with DMSO, palbociclib, or the three combination treatments. GSEA analysis indicated that the EMT gene set was consistently upregulated by palbociclib rather than palbociclib plus alpelisib treatment, whereas treatment with palbociclib plus cetuximab or palbociclib plus cisplatin led to inconsistent results across the three cell lines (Additional file 1: Fig. S6b). Considering that the EMT gene set includes two distinct groups of genes representing the epithelial phenotype or mesenchymal phenotype of cancer cells [52], we then separately examined the expression of epithelial-related (*n* = 169) and mesenchymal-related genes (*n* = 48) in combination treatment groups compared with that under palbociclib monotherapy to identify differences in the effects of these combinations on the EMT process in detail.

Notably, palbociclib plus alpelisib induced significant downregulation of mesenchymal-related genes in CAL27 and FADU cells (NES = − 1.85 and − 1.64, *P* = 0.008 and  0.015, respectively, Fig. 5c), thus indicating EMT inhibition. Palbociclib plus cetuximab induced significant upregulation of epithelial-related genes in FADU and HN30 cells (NES = 1.51 and 1.29, *P* = 0.002 and 0.049, respectively) and significant downregulation of mesenchymal-related genes in HN30 (NES = − 1.69, *P* = 0.012, Fig. 5c) also demonstrated similar EMT inhibition. On the contrary, palbociclib plus cisplatin did not result in any obvious inhibitory effects on EMT in all three cell lines and indeed appeared to induce a significant decrease in transcription of epithelial-related genes in CAL27 cells (NES = − 1.53, *P* = 0.004) and upregulation of mesenchymal-related genes in FADU cells (NES = 1.58, *P* = 0.014), suggesting the activation of EMT process (Fig. 5c and Additional file 1: Fig. S6c).

Western blot analysis revealed that palbociclib monotherapy induced an increase of mesenchymal markers N-cadherin, Vimentin, and Slug, while the palbociclib plus alpelisib combination attenuated the levels of corresponding mesenchymal markers in all these three cell lines (Fig. 5d and Additional file 1: Fig. S6d). However, the palbociclib plus cetuximab induced a relatively minor reduction in the levels of these markers in FADU and CAL27 cells, which was consistent with the limited effects of this combination on the Mes signature (Fig. 5c). In addition, neither palbociclib plus alpelisib nor palbociclib plus cetuximab elicited any discernible effects on E-cadherin in each cell line, indicating that E-cadherin was not likely required for the upregulation of epithelial gene set (Fig. 5c, d and Additional file 1: Fig. S6d). Notably, palbociclib plus cisplatin did not elicit any detectable effects in any of these mesenchymal markers. Taken together, our results demonstrate that palbociclib plus alpelisib and palbociclib plus cetuximab might enhance the therapeutic effects of palbociclib monotherapy through the blockade of EMT.

### RRM2 can induce EMT to attenuate the synergistic effects of palbociclib combined with alpelisib or cetuximab

Ribonucleotide Reductase Regulatory Subunit M2 (RRM2) has been reported as a therapeutic target via activating EMT in HNSCC and other cancers [53, 54]. Our transcriptomic and Western blot analysis also confirmed that RRM2 was downregulated in HNSCC cells treated with palbociclib plus alpelisib or palbociclib plus cetuximab (Fig. 5d and Additional file 1: Fig. S6d, e). To evaluate the potential contribution of RRM2 in these combination treatments, we generated RRM2 stable overexpression (OE) and empty vector (EV) control lines in the FADU and CAL27 backgrounds (Fig. 5e and Additional file 1: Fig. S6f). The synergistic effects of palbociclib combined with alpelisib or cetuximab were significantly attenuated by RRM2 OE, which were then reversed by RRM2 inhibitor osalmid [55] (Fig. 5f and Additional file 1: Fig. S6g). However, neither RRM2 OE nor osalmid treatment exerted similar effect in cells treated with palbociclib and cisplatin combination (Additional file 1: Fig. S6h). Based on these findings, it was reasonable to speculate that RRM2 functioned specifically in response to the palbociclib plus alpelisib and palbociclib plus cetuximab combinations.

To further understand whether RRM2 OE counteracted the synergistic effects of these two combinations via inducing EMT, we first conducted transwell assays. The results showed that RRM2 overexpression led to increased cell migration and invasion, which could be reversed by osalmid treatment (Additional file 1: Fig. S6i, j). Moreover, the expressions of three mesenchymal markers were upregulated in RRM2-OE cells (Fig. 5e and Additional file 1: Fig. S6f). These findings indicated that RRM2 OE could induce EMT in HNSCC cells, which then led us to further investigate the genetic factors responsible of EMT regulation. We found that the decreases in N-cadherin, Vimentin, and Slug induced by these palbociclib combination treatments in RRM2 EV cells were partially attenuated by RRM2 OE (Fig. 5g and Additional file 1: Fig. S6k). However, we observed a similar downregulation of these genes following co-administration of osalmid with either of the two combinations (Fig. 5g and Additional file 1: Fig. S6k). To sum up, these data indicate that RRM2 overexpression could induce EMT to attenuate the synergistic effects of palbociclib combined with alpelisib or cetuximab.

### Evaluation of palbociclib-based treatment combinations in molecularly defined PDXs

**Fig. 6:**
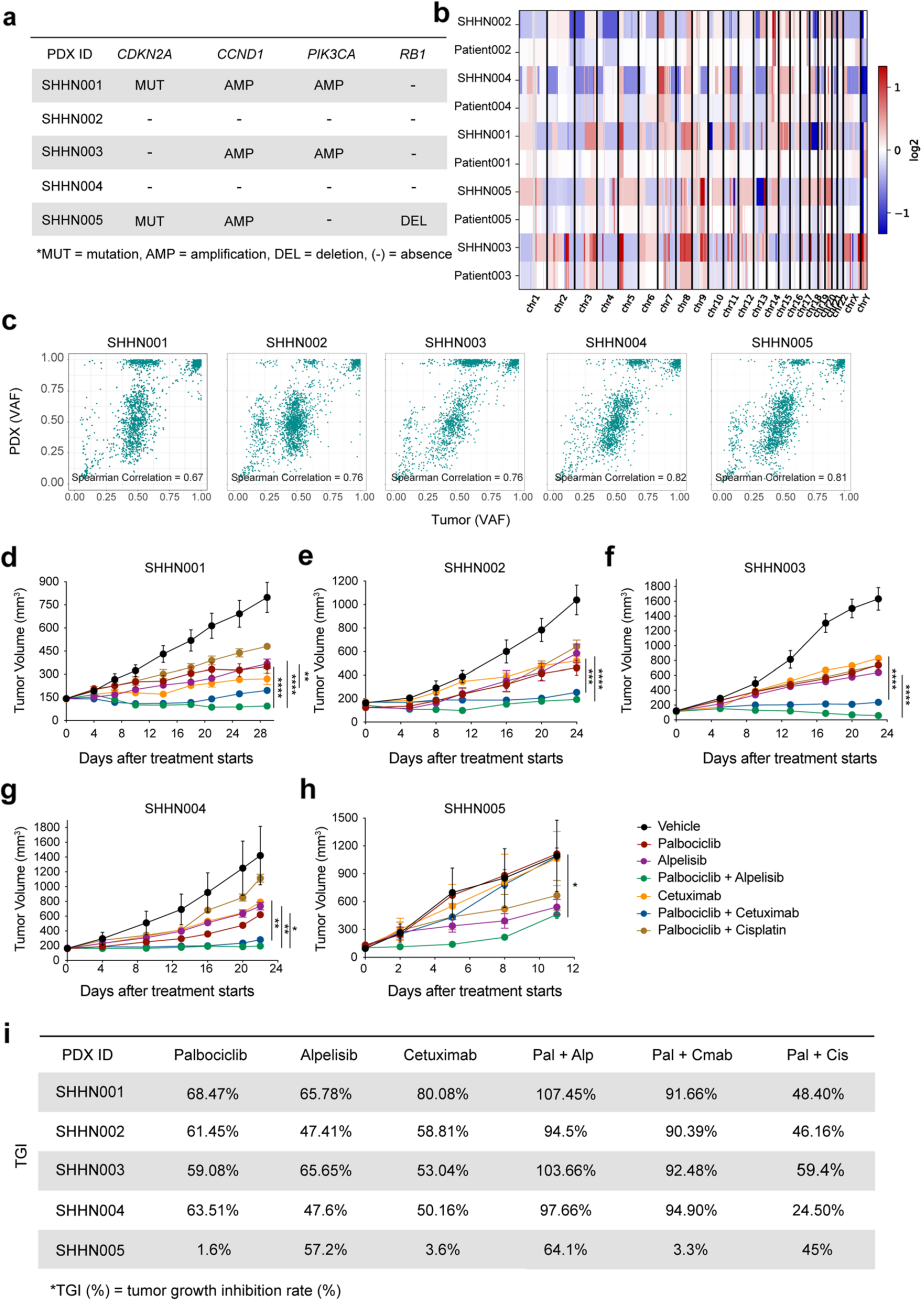
Genetic annotation of the HPV^neg^ HNSCC PDX models and evaluation of selected palbociclib-based treatment combinations in PDX models. **a** Whole exome sequencing was conducted in five selected PDX models. *CDKN2A*, *CCND1*, *PIK3CA*, and *RB1* status of the 5 selected PDX models are shown. **b** Heat map comparing copy number aberrations between matched tumor biopsy and PDX models in a log2 scale. Red colors indicate gains, and blue colors indicate losses. Chr. chromosome. **c** Scatter plots displaying correlations between PDX DNA VAF and patient tumor DNA VAF. The Spearman correlation value is in the lower right-hand corner of each plot. **d–h** Five patient-derived xenograft models from HPV^neg^ HNSCC patients were treated with vehicle (sodium lactate buffer, 50 mmol/L, pH 4.0), palbociclib (60 mg/kg once daily), alpelisib (20 mg/kg once daily), cetuximab (1 mg/kg twice a week), palbociclib plus alpelisib, palbociclib plus cetuximab, and palbociclib plus cisplatin (cisplatin 3 mg/kg once a week) (*n* = 5–10). Tumor growth was measured every other day. Tumor volumes are shown. The data are shown as the mean ± SEM. n.s., not significant; *, *P* < 0.05; **, *P* < 0.01; ***, *P* < 0.001; ***, *P* < 0.0001, as estimated by two-way ANOVA. **i** Summary of TGI values of each treatment in five PDX models. MUT: mutation, AMP: amplification, DEL: deletion, TGI (%): tumor growth inhibition (%)

To validate the synergistic effects of palbociclib-based treatment combinations from a more clinically relevant perspective, palbociclib/alpelisib/cetuximab monotherapy and three combinations mentioned above were further evaluated in five molecularly defined PDX models (Fig. 6a and Additional file 2: Table S7). The CNV landscape and percentage of tumor variants defined by variant allele frequency (VAF) were generally maintained between PDX models and the matched tumors (Fig. 6b, c). The treatment efficacy was evaluated by the percentage of tumor growth inhibition [32], and TGI > 60% was considered meaningful as a significant responder as previously reported [56].

Our results demonstrated that palbociclib monotherapy caused significant tumor inhibition in  three of five selected PDX models (SHHN001, SHHN002, and SHHN004) with TGI = 68.47%, 61.45%, and 63.51%, respectively (Fig. 6d, e, g, i). In particular, SHHN005 showed no response to palbociclib monotherapy (TGI = 1.60%, Fig. 6h, i), in which *CCND1* amplification and *CDKN2A* mutation were found to be concurrent with *RB1* deletion, and the lack of Rb expression was further validated in the xenograft that might explain its resistance (Fig. 6a and Additional file 1: Fig. S7a). Cetuximab monotherapy showed a lack of tumor suppressing effect in four of five PDX models (TGI ranging from 3.60 to 58.81%, SHHN001 TGI = 80.08%, Fig 6d–i). Alpelisib monotherapy exerted significant antitumor efficacy in the *PIK3CA* amplified SHHN001 and SHHN003, with TGI = 65.78%, 65.65% (Fig. 6d, f, i). Though not defined as significant responders, partial antitumor effect was observed in the other three models (TGI ranging from 47.41 to 57.20%, Fig. 6e, g–i). Thus, palbociclib generally has a better therapeutic effect as monotherapy in Rb-proficient HNSCCs when compared with cetuximab or alpelisib monotherapy.

Palbociclib plus cetuximab showed superior therapeutic effect in three of five PDX models (SHHN002 TGI = 90.39%, SHHN003 TGI = 92.48%, SHHN004 TGI = 94.90%) when compared with cetuximab monotherapy (*P* = 0.003, *P* < 0.0001, *P* = 0.0061, Fig. 6e–g), and in three PDX models (SHHN001 TGI = 91.66%, SHHN002 TGI = 90.39%, and SHHN003 TGI = 92.48%) when compared with palbociclib monotherapy (*P* = 0.0459, *P* = 0.0066, *P* < 0.0001, Fig. 6d–f). More importantly, palbociclib plus alpelisib had superior therapeutic effect in four of five evaluated PDXs (except for the Rb-deficient SHHN005) when compared with alpelisib monotherapy (Fig. 6e–g), and in all five PDX models when compared with palbociclib monotherapy (Fig. 6d–h). Notably, tumor regression (TGI > 100%) was only observed in palbociclib plus alpelisib-treated groups in two *PIK3CA*-amplified models, SHHN001 and SHHN003 with a TGI = 107.45% and 103.66%, respectively (Fig. 6d, f).

No significant synergistic effect was observed in palbociclib plus cisplatin combination in all five evaluated PDXs (Fig. 6d–h). Of note, palbociclib plus cisplatin was found with a reduced therapeutic effect when compared with palbociclib monotherapy in SHHN001 (TGI = 48.40% vs TGI = 68.47%), SHHN002 (TGI = 46.16% vs TGI = 61.45%), and SHHN004 (TGI = 24.50% vs TGI = 63.51%, Fig. 6d, e, g). Consistent with the drug screening results from the cell line experiments, palbociclib plus cetuximab or alpelisib targeted treatment combination showed superior therapeutic effects when compared with palbociclib plus cisplatin treatment in all 5 PDX models (Fig. 6d–h). Notably, all treatment combinations were well-tolerated in mice with no significant reductions in bodyweight in all models (Additional file 1: Fig. S7b), as well as no obvious hematologic aberrations or organ lesions (Additional file 1: Fig. 7c, d). Taken together, our in vivo studies using five PDX models with heterogeneous genetic backgrounds provide further evidence that palbociclib can synergize with PI3K and EGFR inhibition and suggest that patients harboring *PIK3CA* alterations are more likely to benefit from these treatment combinations.

## Discussion

Our study aims to identify different ways to improve the efficacy of CDK4/6 inhibitors in HPV^neg^ HNSCC. By using palbociclib-based combination drug screening, we found that alpelisib, a PI3K inhibitor, exerted potent synergistic effects when combined with palbociclib. Notably, this combination strategy was illustrated to have a higher synergism than palbociclib combined with cetuximab or cisplatin, both of which have been evaluated in phase II clinical trials. Additionally, this increased efficacy was particularly evident in cases with *PIK3CA* alterations. Thus, our study adds to the growing number of rational combinations with CDK4/6 inhibitors in molecularly defined patients that could fulfill the promise of targeting cell-cycle pathway in molecularly defined patients in the clinic.

High-throughput drug screening in a matrix is a pivotal strategy exploring drug-drug pairs for potential synergy use. This approach led to the discovery of novel, highly effective combination options for osimertinib-based and ibrutinib-based therapeutics [18, 57]. However, few studies have systematically evaluated the synergistic potential of CDK4/6 inhibitors with other therapeutic agents. Here, we conducted a palbociclib-based combination matrix screening and identified PI3K pathway, EGFR, and MEK inhibitors containing 75% of the top 20 palbociclib-based synergistic drugs. Notably, the therapeutic efficacy of palbociclib plus selective PI3K pathway inhibitors was also investigated preclinically or in clinical trials among other solid tumors (e.g., breast cancer [58, 59], oral squamous cell carcinoma [60], and hepatocellular carcinomas [61]), supporting our conclusion that PI3K inhibitors were promising synergistic agents for palbociclib-based treatment. In addition to PI3K inhibitors, we also observed that mTOR and AKT inhibitors showed relatively well average ExcessHSA in the palbociclib combinations, which has also been reported by other researchers in HNSCC [60].

Activation of PI3K pathway is a frequent observed in hallmark of cancer, which is highlighted by the prevalence of somatic mutations or amplification of *PIK3CA* in HNSCC. Our results demonstrated that PI3K inhibitors showed prominently therapeutic efficacy in *PIK3CA*-altered HPV^neg^ HNSCC while CDK4/6 inhibition has also been proven to improve the response to PI3K inhibitors in *PIK3CA-*mutated breast cancers [62]. Interestingly, a PI3K inhibitor-based combination drug screening conducted on *PIK3CA* mutant breast cancer revealed that combined CDK4/6-PI3K inhibition could overcome intrinsic and acquired resistance of PI3K inhibitors and lead to tumor regressions [63]. Recently, alpelisib was approved by the FDA as the first PI3Kα inhibitor for the treatment of HR-positive, HER2-negative, *PIK3CA*-mutated, advanced or metastatic breast cancer patients [64]. Triple PI3K:CDK4/6:ER combination therapy was also proposed to be evaluated to delay and/or prevent the acquired resistance of CDK4/6-based therapy [65–67]. Additionally, preclinical studies and clinical trials demonstrated the safety and combinatory potential of alpelisib with cisplatin, cetuximab, paclitaxel, or docetaxel in HNSCC (NCT02145312, NCT01602315, and NCT02051751). To sum up, alpelisib, as a representative PI3K inhibitor, was demonstrated to be a promising synergistic drug for the treatment of HPV^neg^ HNSCC.

We also observed other drugs with high average ExcessHSA in HNSCC cell lines that represented potentially valuable combination strategies. For example, appreciable synergistic effects were induced in 3 out of 4 cell lines after combining palbociclib with the CDK2 inhibitor, milciclib. Importantly, Cyclin E-CDK2 complexes drive cell-cycle progression (S-phase entry) and the activation of CDK2 represents one of the mechanisms of CDK4/6i resistance in breast cancer [68]. In addition, we also found two inhibitors (JQ1 and I-BET-762) targeting BRD4, one of the bromo- and extra-terminal domain (BBD) proteins, which ranked 2nd and 18th in the 6 × 6 screening that represents another promising combined therapeutic strategy. BBD proteins are emerging therapeutic targets in TNBC [69] and able to elicit synthetic lethal with several genes including CDK4 [70]. Nevertheless, it is absolutely necessary to refine the potential biomarkers used in combinational treatment to ensure highly efficient responses.

EMT is a well-studied complex biological process in embryonic development that is recapitulated during tumor progression, metastasis, and drug resistance [68]. Previous studies have demonstrated that palbociclib treatment promoted or inhibited EMT in different types of cancer [46, 71]. Here, we found that EMT gene set was upregulated by palbociclib while two targeted treatment combinations exerted significant synergistic effects through suppression of the EMT-related impacts induced by palbociclib. The synergistic effect of these two combinations was attenuated by RRM2 overexpression and restored after subsequent treatment with osalmid, an RRM2 inhibitor. Recently, the oncogenic role of RRM2 has been linked to the EMT process in esophageal adenocarcinoma, glioma, and prostate cancers [53, 54, 72] and multiple RRM2 inhibitors have been developed and are being investigated in clinical trials as monotherapy or combinational treatment option. In particular, RRM2 inhibitors was found to be highly effective when combined with cell-cycle checkpoint inhibitors in Ewing sarcoma [73]. Thus, further studies are warranted to fully explore the potential of such promising therapeutic combinations. 

## Conclusions

In summary, we provided a comprehensive palbociclib-based drug-drug interaction dataset and identified alpelisib (a PI3Kα inhibitor) as the most potent synergistic agent in HPV^neg^ HNSCCs, with particularly higher efficacy in cases with *PIK3CA* alterations. Additionally, by using a panel of genetically diverse cell lines and PDX models, we found that palbociclib plus alpelisib outperformed palbociclib plus either cetuximab or cisplatin, which are currently being investigated in clinical trials in HNSCC. Thus, the examination of palbociclib-based drug combinations provides insights into the systematic combinatory effects associated with CDK4/6 inhibition and supports further exploration of biomarker-guided clinical trials using palbociclib plus alpelisib combination in HPV^neg^ HNSCC.

## Supplementary Information

**Additional file 1: Supplementary Figure 1.** HPV^neg^ HNSCC models showed a limited response to CDK4/6 inhibitor palbociclib monotherapy, related to Fig. 1. **Supplementary Figure 2.** 10 × 10 matrix screening of palbociclib combined with four PI3K inhibitors in HPV^neg^ HNSCC cell lines, related to Fig. 2. **Supplementary Figure 3.** Assessment of palbociclib-based combinational therapy with four PI3K inhibitors in HPV^neg^ HNSCC cell lines, related to Fig. 3. **Supplementary Figure 4.** Assessment of abemaciclib-based combinational therapy with four PI3K inhibitors in HPV^neg^ HNSCC cell lines, related to Fig. 3. **Supplementary Figure 5.** GSEA and western blot analyses of palbociclib-alpelisib combined therapy in HPV^neg^ HNSCC cell lines, related to Fig. 4. **Supplementary Figure 6.** Effect and mechanism of selected drug combinations in HPV^neg^ HNSCC cell lines, related to Fig. 5. **Supplementary Figure 7.** Information of relevant in vivo experiments using HPV^neg^ PDX models, related to Fig. 6.

**Additional file 2: Table S1.** Information of 162 chemicals. **Table S2.** Epithelial and Mesenchymal Gene lists. **Table S3.** Primers in this study. **Table S4.** Primary antibodies. **Table S5.** Genetic annotation of cell lines in this study. **Table S6.** Average ExcessHSA scores of all compounds. **Table S7.** Genetic annotation of PDX models.

## Data Availability

The datasets of Cancer Cell Line Encyclopedia and Genomics of Drug Sensitivity in Cancer used in the analysis are publicly available from figshare, 10.6084/m9.figshare.11791698.v3 and European Genome-phenome Archive (EGA), https://ega-archive.org/studies/EGAS00001000978, respectively. The raw sequence data in this study have been deposited in the Genome Sequence Archive in BIG Data Center, Beijing Institute of Genomics (BIG), Chinese Academy of Sciences, under accession number HRA002069 and HRA002070. The Web link for these publicly available data (with controlled access on reasonable request) is https://ngdc.cncb.ac.cn/gsa-human.

## Abbreviations

ATCC: American type culture collection
BCA: The bicinchoninic acid
CCK8: Cell counting kit 8
CDK4/6: Cyclin-dependent kinase 4/6
CNV: Copy number variants
DMSO: Dimethylsulfoxide
EGFR: Epidermal growth factor receptor
EMT: Epithelial mesenchymal transition
ER: Estrogen receptor
ExcessHSA: Excess highest single agent
GSEA: Gene set enrichment analysis
HER2: Human epidermal growth factor receptor 2
HNSCC: Head and neck squamous cell carcinoma
HPV^neg^: Human papillomavirus negative
IC50: Half-maximal inhibitory concentration
PD-1: Programmed death-1
PDX: Patient-derived xenografts
PVDF: Polyvinylidene difluoride
RB/Rb: Retinoblastoma
RRM2: Ribonucleotide Reductase Regulatory Subunit M2
TGI: Tumor growth inhibition
VAF: Variant allele frequency
