# Supplementary material for: Palbociclib-based high-throughput combination drug screening identifies synergistic therapeutic options in HPV-negative head and neck squamous cell carcinoma
Source: BMC Med. 2022 May 12;20:175. doi: 10.1186/s12916-022-02373-6 (PMC9097351; doi:10.1186/s12916-022-02373-6)
Supplement: Supplementary file 1 — Additional file 1: Supplementary Figure 1. HPVneg HNSCC models showed a limited response to CDK4/6 inhibitor palbociclib monotherapy, related to Fig. 1. Supplementary Figure 2. 10 × 10 matrix screening of palbociclib combined with four PI3K inhibitors in HPVneg HNSCC cell lines, related to Fig. 2. Supplementary Figure 3. Assessment of palbociclib-based combinational therapy with four PI3K inhibitors in HPVneg HNSCC cell lines, related to Fig. 3. Supplementary Figure 4. Assessment of abemaciclib-based combinational therapy with four PI3K inhibitors in HPVneg HNSCC cell lines, related to Fig. 3. Supplementary Figure 5. GSEA and western blot analyses of palbociclib-alpelisib combined therapy in HPVneg HNSCC cell lines, related to Fig. 4. Supplementary Figure 6. Effect and mechanism of selected drug combinations in HPVneg HNSCC cell lines, related to Fig. 5. Supplementary Figure 7. Information of relevant in vivo experiments using HPVneg PDX models, related to Fig. 6. [file 12916_2022_2373_MOESM1_ESM.docx]

**Supplementary Figures**

**Supplementary Figure 1 (a-d)**

**Supplementary Figure 1 (e-h)**

**
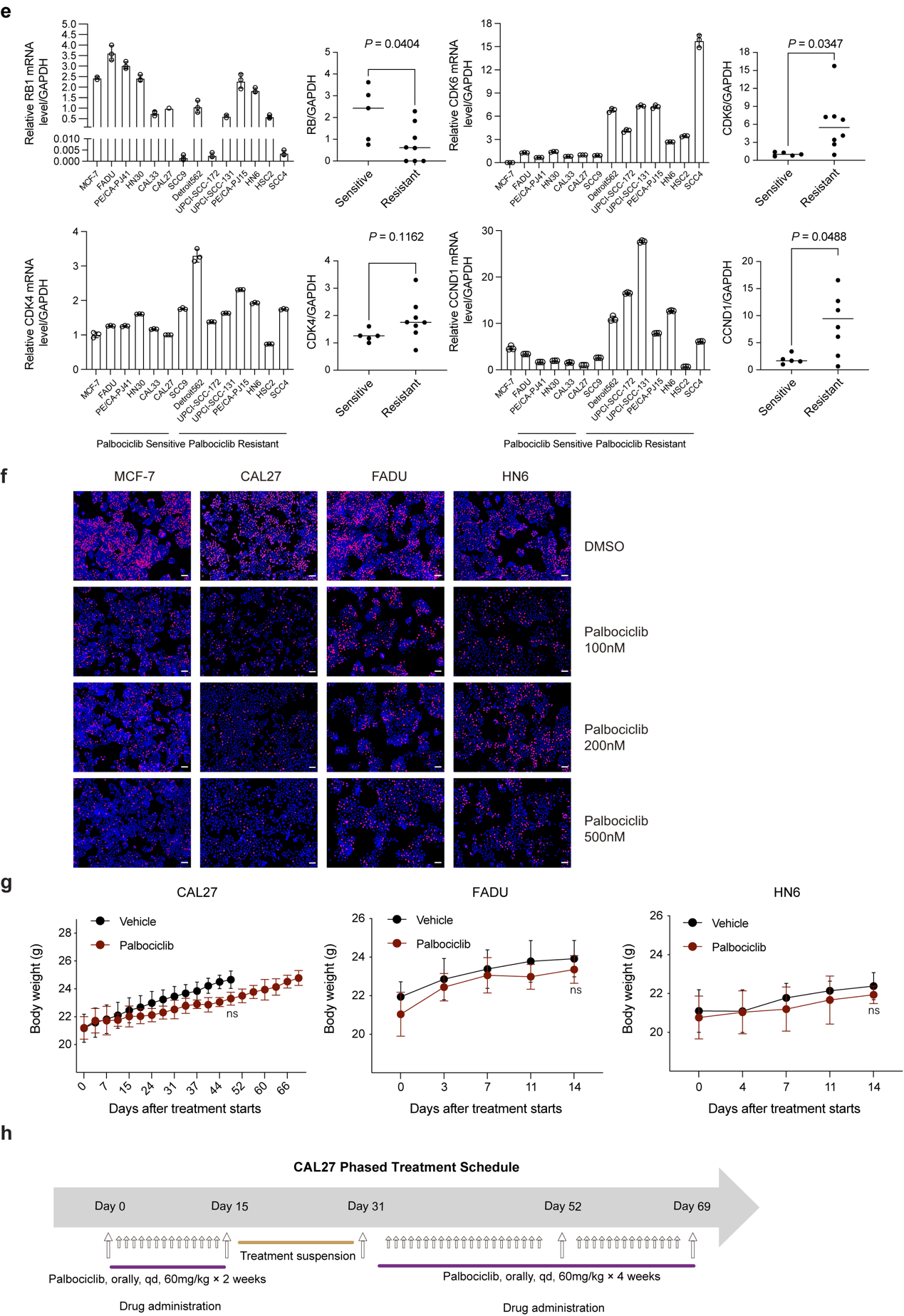
**

**Supplementary Figure 1. HPV^neg^ HNSCC models showed a limited response to CDK4/6 inhibitor palbociclib monotherapy, related to Figure 1**

**a** Dose-response curves of cell viability of 14 cell lines shown in Figure 1a, as measured by CCK-8 and compared to DMSO control after 72-hour exposure to palbociclib (left) (n = 3 biological replicates; mean ± SD). Box plot shows half maximal inhibitory concentration (IC_50_) values (mean ± SD) of palbociclib in 14 cell lines (right). **b** Mutation concordance of well-known oncogenes (e.g., *TP53*, *PIK3CA*, *CDKN2A*) in overlapped 7 HPV^neg^ HNSCC cell lines among our cell line panel, Cancer Cell Line Encyclopedia and Genomics of Drug Sensitivity in Cancer. **c** Scatter dot plots show comparison of related protein expression of each gene in PS group and PR group. *P* values were calculated using Student’s *t*-test. **d** Scatter dot plots show IC_50_ of palbociclib in 13 HPV^neg^ HNSCC cell lines with *CDKN2A* deletion/mutation or with no alteration, with *CCND1* amplification or no CNV, and with *CDK6* amplification or no CNV. *P* values were calculated using Student’s *t*-test. **e** qRT PCR analysis to determine mRNA expressions of cell-cycle related genes shown in Figure 1b, with comparison of related mRNA expression of each gene in PS group and PR group. *P* values were calculated using Student’s *t*-test. **f** EdU staining photographs of the cell lines listed in Figure 1C after 100, 200 and 500 nM palbociclib treatment for 48 hours. Scale bar = 100 μm. **g** Body weight of HNSCC cell lines injected mice treated with palbociclib monotherapy. The data are shown as the mean ± SD. n.s., not significant as estimated using a two-way ANOVA. **h** Schema of *in vivo* experiment of CAL27 xenografts under palbociclib treatment.**Supplementary Figure 2**

**Supplementary Figure 2. 10🞨10 matrix screening of palbociclib combined with four PI3K inhibitors in HPV^neg^ HNSCC cell lines, related to Figure 2**

**a** Schematic diagram of the high-throughput combination drug screening. **b** Histogram showing the distribution of Z-prime score from 648 screening 96-well plates. **c** Heat maps of 10🞨10 % Response and ∆Bliss for combinational therapy using palbociclib with pictilisib, alpelisib, taselisib (GDC-0032) or apitolisib, in HN6, CAL27 and, SCC9 cell lines. **d** ExcessHSA scores in 10×10 matrix screening of combinational palbociclib treatment with the four PI3K inhibitors shown in four cell lines (FADU, HN6, CAL27 and SCC9). The data are shown as the mean ± SD. n.s., not significant; * *P* < 0.05, as estimated using a two-way ANOVA.

**Supplementary Figure 3 (a-c)**

**Supplementary Figure 3 (d-f)**

**Supplementary Figure 3 (g-k)**

**Supplementary Figure 3. Assessment of palbociclib-based combinational therapy with four PI3K inhibitors in HPV^neg^ HNSCC cell lines, related to Figure 3.**

**a** Dose response curves of cell viabilities of palbociclib (left) with top candidate therapeutic agents apitolisib (100 nM) and pictilisib (100 nM), in eight HNSCC cell lines (red, *PIK3CA* mut/amp; blue, *PIK3CA* WT). **b-e** Dose-response curves of cell viabilities of gradient concentrations of palbociclib (blue), PI3K inhibitors (red), or the two drugs combined (purple), including palbociclib and alpelisib (**b**), palbociclib and GDC-0032 (**c**), palbociclib and apitolisib (**d**), palbociclib and pictilisib (**e**), for 72 hours in *PIK3CA* mut/amp and *PIK3CA* WT HNSCC cell lines. **f-h** Cell proliferation estimated by EdU analysis after treatment with DMSO vehicle (control; gray), palbociclib (blue), GDC-0032, apitolisib, or pictilisib (red), and two-drug combinations (purple), including palbociclib and GDC-0032 (**f**), palbociclib and apitolisib (**g**), palbociclib and pictilisib (**h**), for 48 hours (left). Population of cells at the G0/G1 phase of the cell-cycle measured by flow cytometry after implementing the same treatment as described above (right). **i** The body weights of mice were measured during the treatment process. **j-k** Tumor weight (**j**) and corresponding photographs (**k**) of FADU xenografts after palbociclib, alpelisib and combined therapy. (a-j) The data are shown as the mean ± SD. n.s., not significant; * *P* <0.05, ** *P* < 0.01, *** *P* <0.001, **** *P* < 0.0001, as estimated using a two-way ANOVA.

**Supplementary Figure 4 (a-c)**

**Supplementary Figure 4 (c-d)**

**
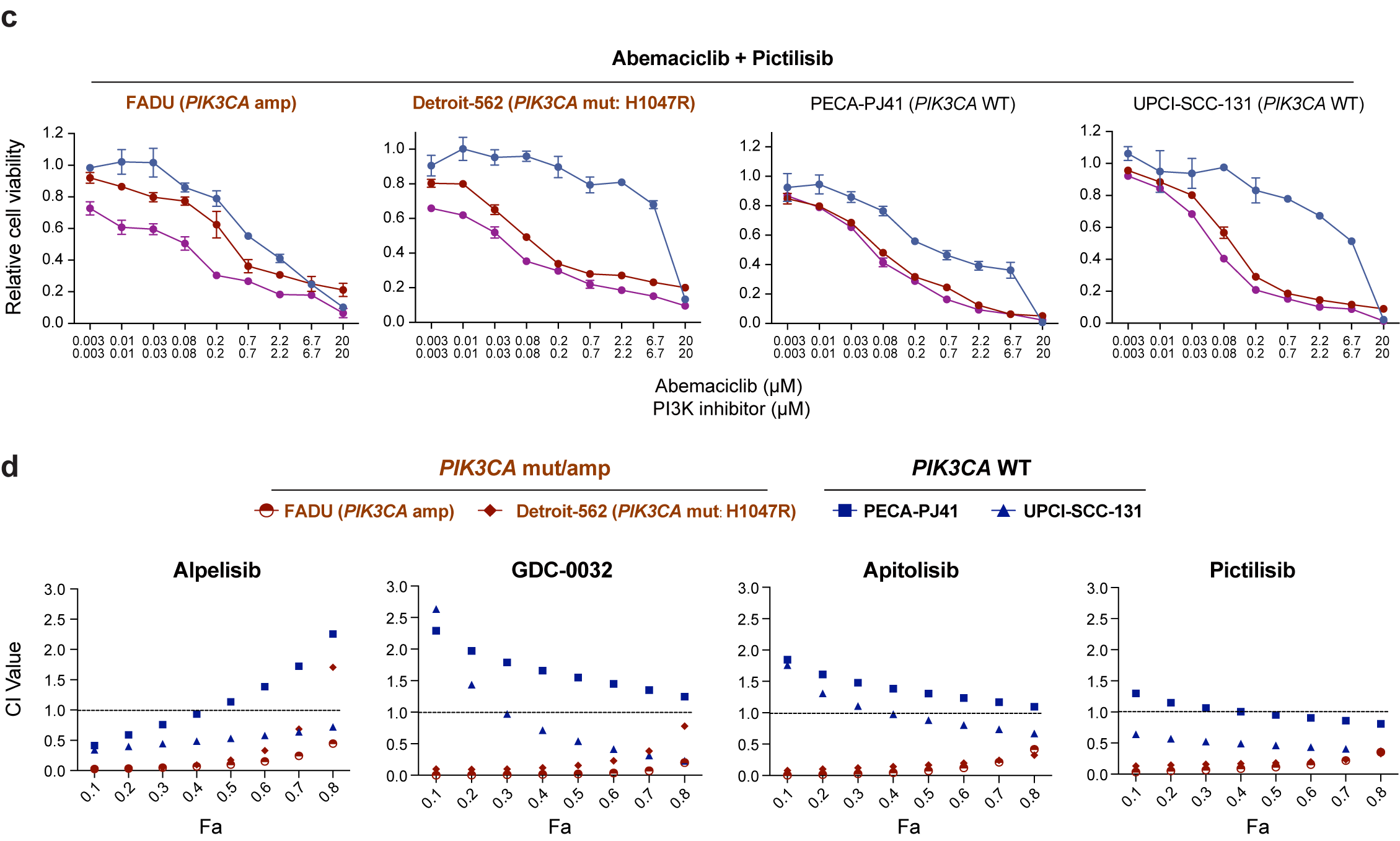
**

**Supplementary Figure 4. Assessment of abemaciclib-based combinational therapy with four PI3K inhibitors in HPV^neg^ HNSCC cell lines, related to Figure 3.**

**a** Dose-response curves of cell viabilities of 14 cell lines shown in Figure 1a, as measured by CCK-8 analysis and compared to DMSO control after 72-hour exposure to abemaciclib (left) (n = 3 biological replicates; mean ± SD). Box plot shows IC_50_ values (mean ± SD) of abemaciclib in 14 cell lines (right). **b** Cell viability of abemaciclib (left) with alpelisib (500 nM), GDC-0032 (50 nM), apitolisib (100 nM) and pictilisib (100 nM), in two *PIK3CA* mut/amp (red) and two *PIK3CA* WT (blue) HNSCC cell lines. **c** Dose-response curves of cell viabilities of gradient concentrations of abemaciclib (blue), PI3K inhibitors (red), or the two drugs combined (purple) for 72 hours in two *PIK3CA* mut/amp (FADU: amp, Detroit-562: mut) and two *PIK3CA* WT (PECA-PJ41, UPCI-SCC-131) HNSCC cell lines. **d** Fa-CI dot plots show the CI values used to identify drug synergies between abemaciclib and four PI3K inhibitors in all 4 experimentally examined HNSCC cell lines from each combination (red, *PIK3CA* mut/amp; blue, *PIK3CA* WT). Horizontal dashed line indicates CI = 1. Values below this threshold indicate drug synergy, while those above it represent drug antagonism. Fa (Fraction affected), CI (combination index).

**Supplementary Figure 5**

**
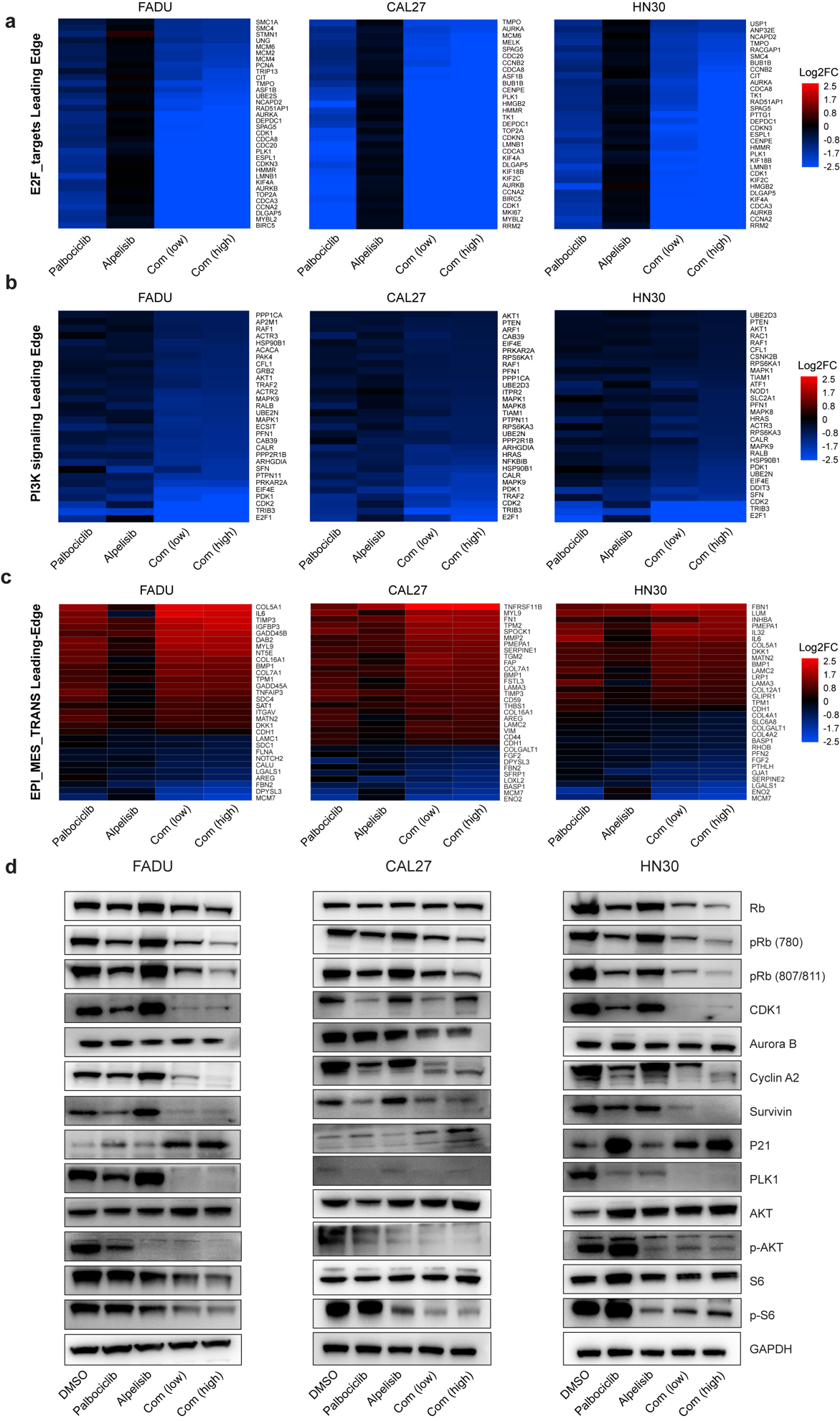
**

**Supplementary Figure 5. GSEA and Western blot analysis of palbociclib-alpelisib combined therapy in HPV^neg^ HNSCC cell lines, related to Figure 4.**

**a-c** The “leading-edge” genes from the HALLMARK_E2F gene sets (**a**), the HALLMARK_PI3K gene sets (**b**) and the HALLMARK-EPI_MES_TRANS gene sets (**c**) were ranked based on the high-dosage combined therapy. **c** Representative Western blot analysis of the indicated E2F targets and PI3K signaling markers are performed in three cell lines treated with palbociclib, alpelisib, and combined therapy.

**Supplementary Figure 6 (a-d)**

**Supplementary Figure 6 (e-k)**

**
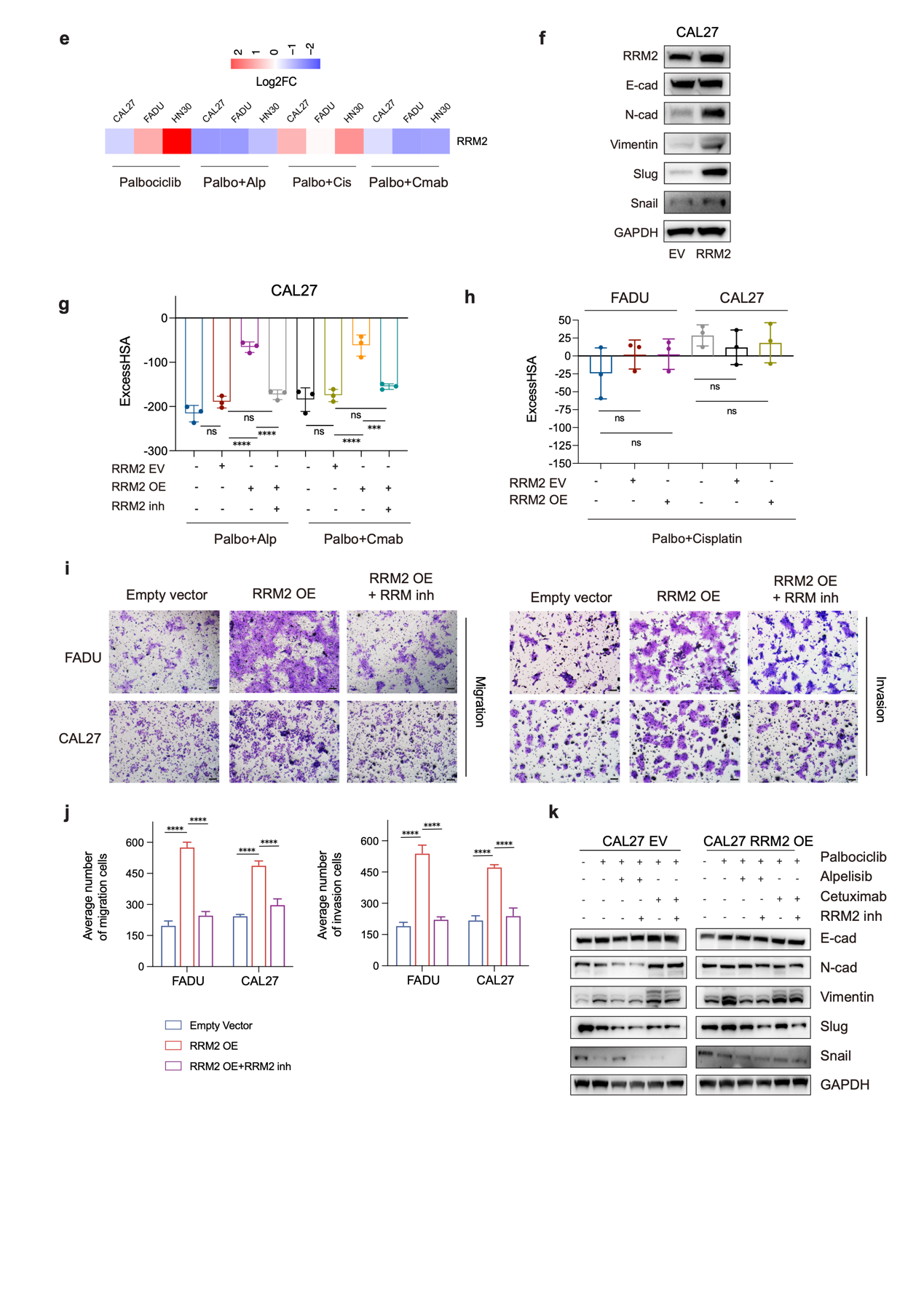
**

**Supplementary Figure 6. Effect and mechanism of selected drug combinations in HPV^neg^ HNSCC cell lines, related to Figure 5.**

**a** % Response and ∆Bliss heat maps for palbociclib combined with alpelisib, cisplatin, or cetuximab. **b** The Hallmark gene sets in HNSCC cell lines treated with palbociclib are listed based on the normalized enrichment scores (NES) estimated using unsupervised hierarchical clustering. **c** GSEA of EMT_Epi and EMT_Mes gene-sets after three combination treatments in three HNSCC cell lines. **d** Western blot analysis of canonical EMT markers in CAL27 and HN30 cells treated with palbociclib, palbociclib plus alpelisib (low dosage), palbociclib plus alpelisib (high dosage), palbociclib plus cisplatin, and palbociclib plus cetuximab. **e** Log2 fold changes in RRM2 mRNA expression in HNSCC after palbociclib monotherapy and three different combination treatments. **f** EMT markers expression detected by Western blot in EV- and RRM2-expressing CAL27 cells. **g** ExcessHSA scores in combination (palbociclib plus alpelisib and palbociclib plus cetuximab)-treated cells for 72 hours compared to DMSO controls in the presence/absence of RRM2 inhibitor osalmid in both EV- and RRM2-expressing CAL27 cells. **h** ExcessHSA scores in EV- and RRM2-expressing CAL27 and FADU cells treated with palbociclib plus cisplatin for 72 hours. The data are shown as the mean ± SD. n.s., not significant; *** *P* <0.001, **** *P* < 0.0001, as estimated using a two-way ANOVA. **i** Cell migration and invasion assays were performed with or without RRM2 inhibitor osalmid in EV- and RRM2-expressing CAL27 and FADU cells. Scale bar = 100 μm. **j** Statistical analysis of average cell migration and invasion cell numbers in different groups in CAL27 and FADU cells. **k** EMT markers expression in both EV- and RRM2-expressing CAL27 cells after palbociclib monotherapy and combined treatment in the presence/absence of RRM2 inhibitor osalmid.

**Supplementary Figure 7 (a-b)**


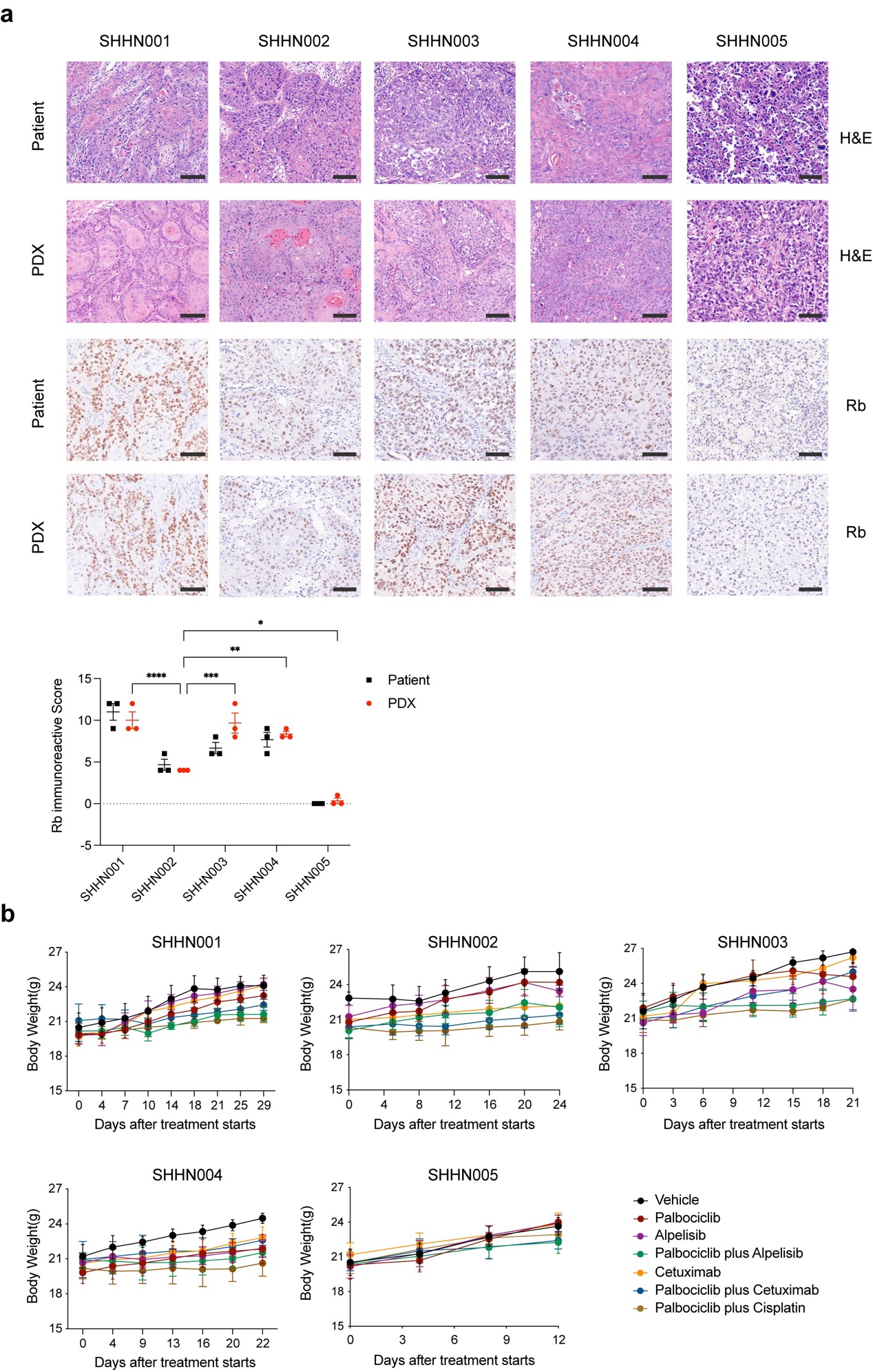


**Supplementary Figure 7 (c-d)**


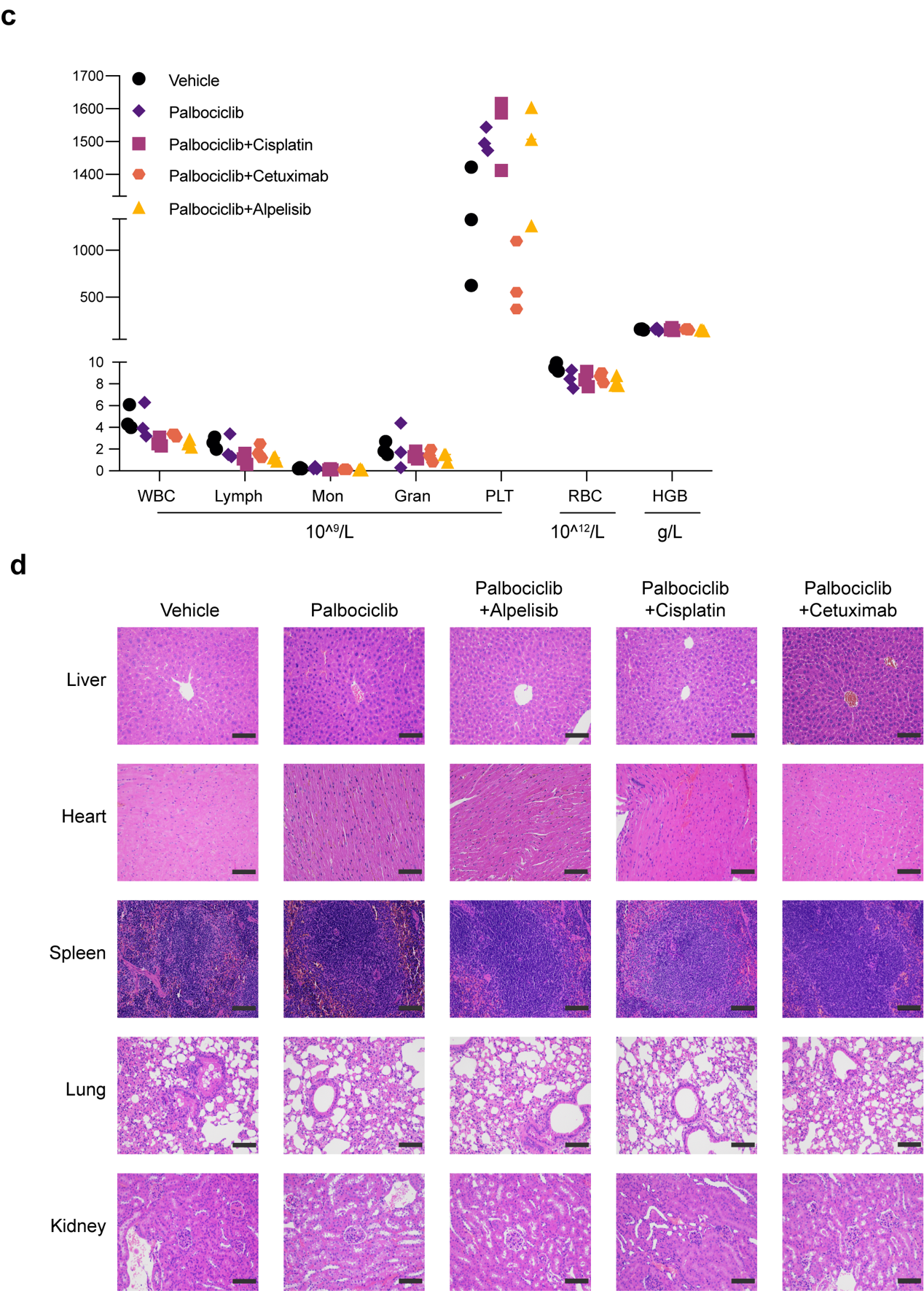


**Supplementary Figure 7. Information of *in vivo* experiments using HPV^neg^ PDX models, related to Figure 6.**

**a** Hematoxylin and eosin (H&E) and IHC staining of Rb protein in PDX models and corresponding patient tumors (Top). Scale bars, 50 μm. Statistical analysis of immunoreactive score of Rb expression in five PDX models and corresponding patient tumors (Bottom). **b** Body weight of 5 PDX models treated with palbociclib monotherapy and combination therapies. **c** Hematological examination of whole blood samples obtained from the PDX models. **d** H&E staining of internal organs from the PDX models. Scale bars, 100 μm.
